# A 2-pyrone-4,6-dicarboxylic acid biosensor for lignin valorization based on a newly identified transcriptional regulatory system

**DOI:** 10.1186/s13068-026-02797-9

**Published:** 2026-07-23

**Authors:** Kenta Tanatani, Hyo Suzuki, Masaya Fujita, Takuma Araki, Yuzo Suzuki, Tsuyoshi Michinobu, Yuichiro Otsuka, Masaya Nakamura, Eiji Masai, Naofumi Kamimura

**Affiliations:** 1 Department of Materials Science and Bioengineering, Nagaoka University of Technology, Nagaoka, Niigata 940-2188 Japan; 2 Department of Forest Resource Chemistry, Forestry and Forest Products Research Institute, Tsukuba, Ibaraki Japan; 3 Department of Materials Science and Engineering, Institute of Science Tokyo, Meguro-Ku, Tokyo Japan

**Keywords:** Lignin valorization, Biosensor, 2-Pyrone-4,6-dicarboxylic acid (PDC), *Sphingobium lignivorans* SYK-6, Biological funneling, High-throughput screening

## Abstract

**Background:**

Lignin is the most abundant renewable aromatic resource on Earth, yet its efficient conversion into value-added chemicals through biological funneling remains a key challenge. *Sphingobium lignivorans* SYK-6 exhibits broad metabolic capacity for lignin-derived aromatic compounds, ranging from monomers to dimers, making it a suitable host for biological funneling. Production systems for the polymer building block 2-pyrone-4,6-dicarboxylic acid (PDC) from lignin-derived aromatic compounds have been constructed using SYK-6. In this study, we developed a PDC-responsive biosensor to facilitate high-throughput screening of improved PDC-producing strains.

**Results:**

PDC-assimilating bacteria were isolated from environmental samples, and their genomes were analyzed to obtain candidate PDC-responsive transcriptional regulatory systems. Among these, the LysR-type transcriptional regulator PdcR and its target promoter P_PDC_ from *Cupriavidus* sp. 8B were identified and characterized as a PDC-responsive system, in which PdcR is suggested to act as both a repressor and an activator. A reporter plasmid pCup1 carrying these components was introduced into a PDC hydrolase gene-deficient strain (Δ*ligI*) of SYK-6 to construct the PDC biosensor Δ*ligI*(pCup1). The biosensor was specific for PDC, with no response observed for its upstream metabolites. When vanillic acid or protocatechuic acid was supplied as a substrate, concentration-dependent fluorescence responses were observed over a range of 0.1–5 mM, demonstrating that PDC production from lignin-derived aromatic compounds can be monitored. Fluorescence increased synchronously with PDC accumulation, suggesting real-time monitoring of PDC production. Furthermore, the biosensor responded to PDC produced from aromatic compounds in softwood black liquor extract.

**Conclusions:**

Δ*ligI*(pCup1) enables real-time, specific detection of PDC produced from lignin-derived aromatic compounds, including those in black liquor extract derived from lignocellulosic biomass, demonstrating applicability to screening for lignin valorization. Integration of this biosensor with diverse strain engineering approaches, including random mutagenesis, adaptive laboratory evolution, and metagenomics-based enzyme discovery, would provide a platform for efficient selection of SYK-6 strains with enhanced PDC production capability.

**Graphical Abstract:**

**Figure:**
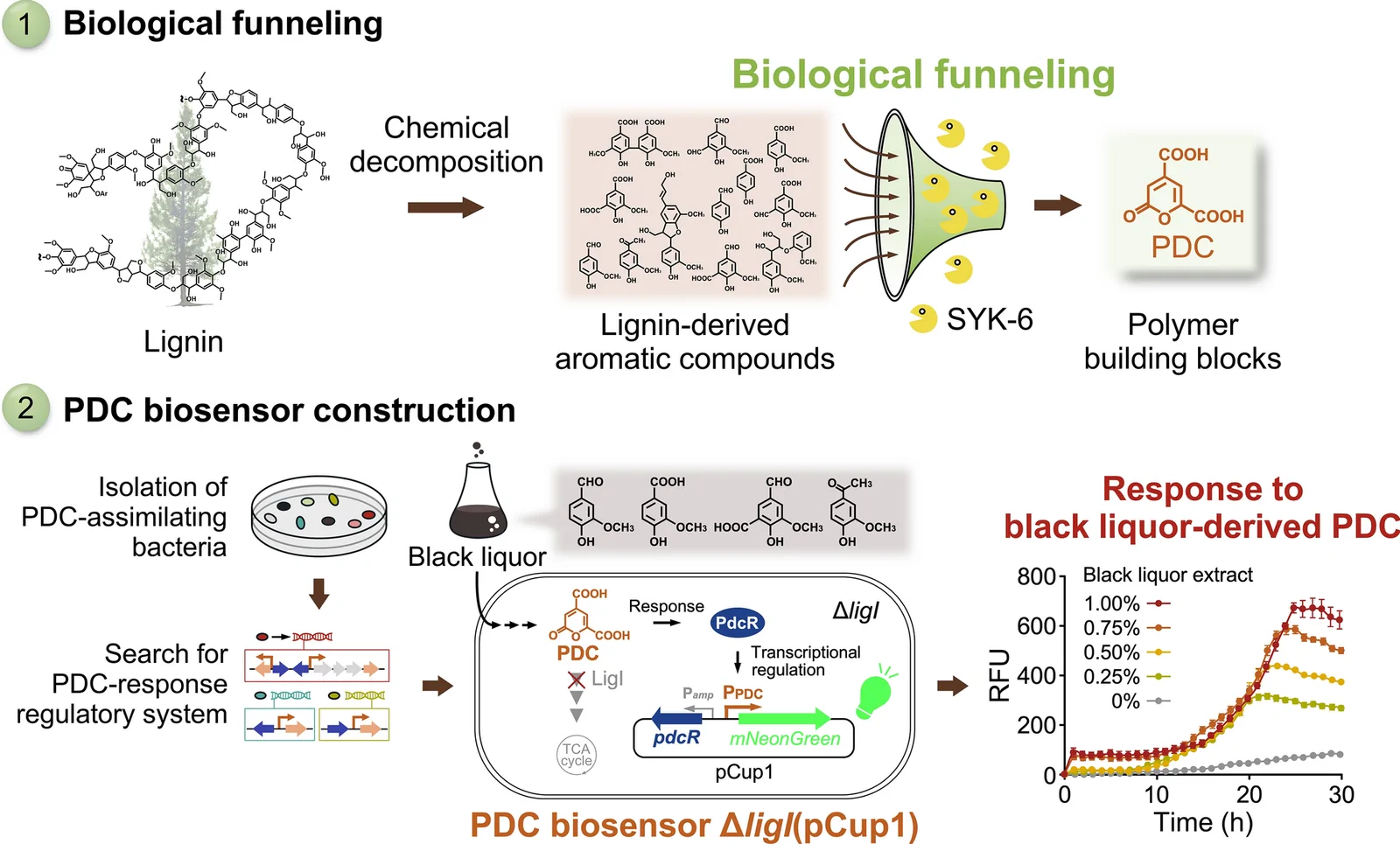

**Supplementary Information:**

The online version contains supplementary material available at https://doi.org/10.1186/s13068-026-02797-9.

## Introduction

Lignin, comprising 15–30% of plant biomass, is the most abundant renewable aromatic polymer on Earth and a promising feedstock for the production of value-added chemicals [1, 2]. Approximately 50 million tons of lignin are generated annually as a byproduct of the pulp and paper industry [2]; however, the vast majority is combusted for energy recovery rather than valorized as a chemical feedstock. Converting lignin into value-added chemicals is therefore a central objective of lignocellulosic biorefinery strategies.

**Fig. 1:**
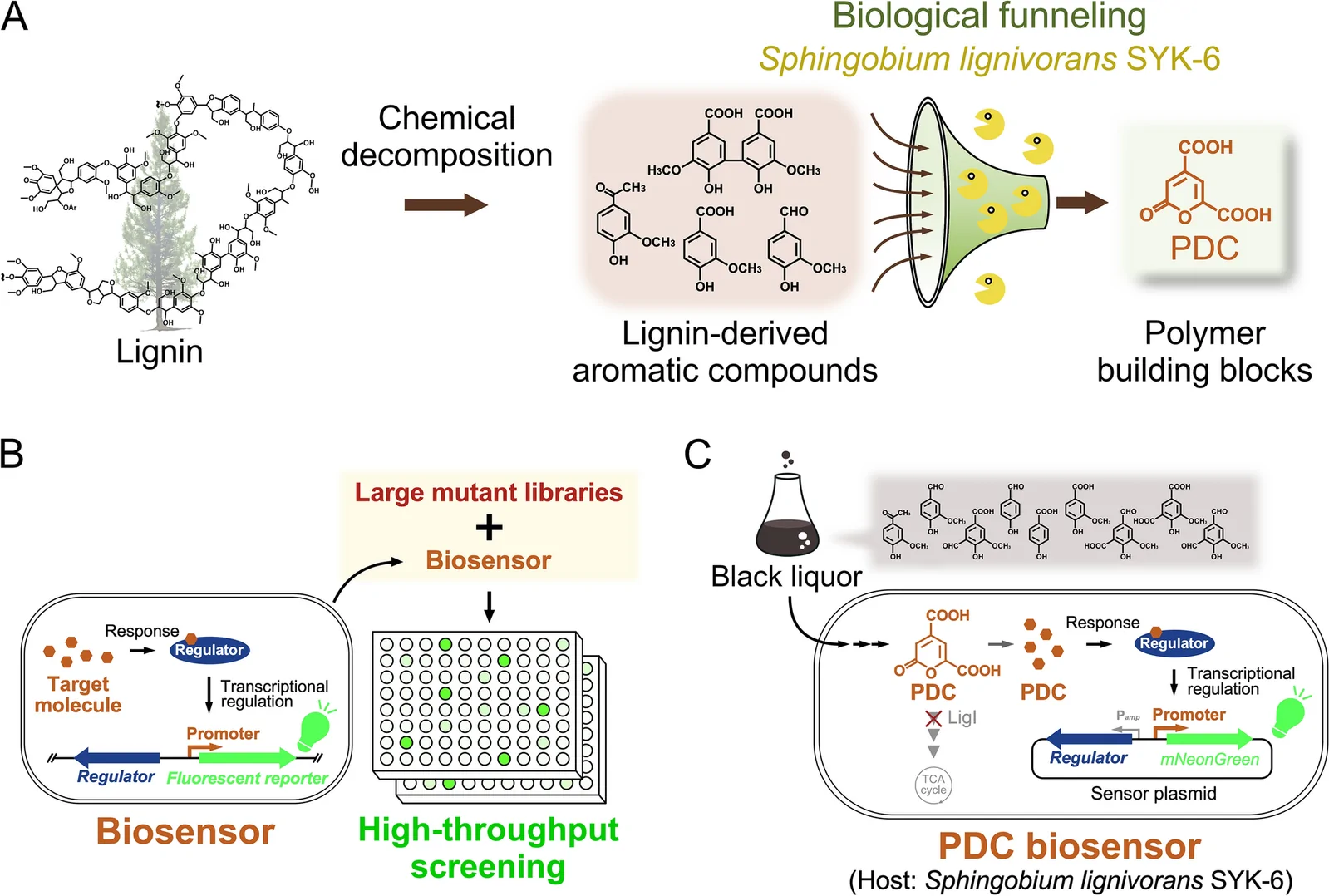
Conceptual overview of this study. **A** Schematic representation of biological funneling using *Sphingobium lignivorans* SYK-6. **B** Transcriptional regulator-based biosensor for high-throughput screening. A transcriptional regulator detects the target molecule (input) and activates expression of a fluorescent reporter gene (output), enabling real-time monitoring of target compound production and fluorescence-based high-throughput screening of large mutant libraries. **C** PDC-responsive biosensor constructed in this study using *S. lignivorans* SYK-6 as the host. Lignin-derived aromatic compounds in black liquor extract are converted to PDC by a PDC hydrolase-deficient strain (Δ*ligI*) of SYK-6, and the accumulated PDC is detected and quantified as a fluorescence signal by the biosensor

To address this challenge, strategies combining chemical depolymerization of lignin with biological funneling into a single target compound have attracted considerable attention [3, 4]. One such target is 2-pyrone-4,6-dicarboxylic acid (PDC), a compound with promising applications as a building block for bioplastics and adhesives, and as a metal chelator [5–7]. Several PDC production systems exploiting metabolic pathways for lignin-derived aromatic compounds have been reported [8–14]. However, when lignocellulosic biomass-derived lignin is used as a feedstock, PDC production efficiency remains well below economically competitive levels. This limitation is partly attributed to the limited capacity of many host bacteria to efficiently convert heterogeneous lignin-derived aromatic compounds, including dimers and oligomers. In this context, *Sphingobium lignivorans* SYK-6, which catabolizes not only monomeric but also dimeric lignin-derived aromatic compounds, represents a promising production platform (Fig. 1A) [15]. Engineered SYK-6 strains lacking the PDC hydrolase gene *ligI* have achieved molar yields of nearly 100% from individual lignin-derived model dimers [16–18]. More recently, PDC was produced at a molar yield of 135% from softwood-derived black liquor [14]. The yield exceeding 100% suggests conversion of unidentified oligomeric components, highlighting the strong potential of SYK-6 as a PDC production host. Achieving economically competitive PDC production will therefore require further improvement of aromatic conversion efficiency in SYK-6. To this end, a high-throughput screening system enabling efficient selection of improved PDC-producing SYK-6 strains is essential.

Transcriptional regulator-based biosensors, constructed by combining a transcriptional regulator, its cognate promoter, and a reporter gene, generate fluorescence signals in response to target compound concentration [19, 20]. Such biosensors have attracted increasing attention as high-throughput alternatives to conventional chromatographic analyses, and their application to screening of large mutant libraries for improved production strains has expanded considerably (Fig. 1B) [21–25]. To apply this approach to the development of improved PDC-producing SYK-6 strains, a biosensor based on a transcriptional regulatory system specifically responsive to PDC is essential. The promoter of the *pmd* operon from *Comamonas* sp. E6 is induced in the presence of PDC [26]. However, this promoter lacks specificity, as it responds to both PDC and protocatechuic acid (PCA), and the corresponding transcriptional regulator has not been identified [26]. To date, no PDC-specific transcriptional regulatory system has been reported, and its identification represents a key challenge in constructing a screening platform for improved SYK-6 strains.

In this study, we developed a PDC-responsive biosensor using *S. lignivorans* SYK-6 as the host to enable screening for improved PDC producers (Fig. 1C). Through genome analysis of PDC-assimilating bacteria isolated from environmental samples, we identified a PDC-specific transcriptional regulatory system from *Cupriavidus* sp. 8B and constructed the biosensor strain Δ*ligI*(pCup1). This biosensor exhibited concentration-dependent fluorescence responses specific to PDC and detected PDC produced from softwood black liquor extract.

## Materials and methods

### Strains, plasmids, culture conditions, and chemicals

The strains, plasmids, and primers used in this study are listed in Additional file 1: Tables S1 and S2. Strains were cultured in Wx minimal medium [27] or in Lysogeny Broth (LB) as the rich medium. Wx medium was supplemented with SEMP (10 mM sucrose, 10 mM glutamate, 0.13 mM methionine, and 10 mM proline) [27]. For reporter assays, Wx-SEMP medium was further supplemented with PDC, PCA, vanillic acid, or black liquor extract as indicated. For solid media, agar was added to a final concentration of 1.5% (w/v). Strains were cultured aerobically at 37 °C for *E. coli* and at 30 °C for all other strains. When required, nalidixic acid (12.5 mg/L) and kanamycin (50 mg/L) were added to SYK-6 cultures, whereas kanamycin (25 mg/L) was added to *E. coli* cultures. PCA and vanillic acid were purchased from Tokyo Chemical Industry Co., Ltd. (Tokyo, Japan). PDC was prepared as described previously [28]. Black liquor extract was prepared from black liquor obtained by oxygen-soda anthraquinone pulping of Japanese cedar [29]. The black liquor was acidified to approximately pH 2.0 with 12 N HCl and extracted three times with an equal volume of ethyl acetate. The organic phase was evaporated under reduced pressure, and the residue was dissolved in dimethyl sulfoxide at half the volume of the original black liquor, yielding a twofold concentrated extract.

### Isolation of PDC-assimilating bacteria

A total of 26 environmental samples, including decaying wood and forest soil, were collected in Niigata and Ibaraki Prefectures, Japan. Each sample was suspended in Wx buffer [12.5 mM KH_2_PO_4_, 27.4 mM Na_2_HPO_4_, and 7.6 mM (NH_4_)_2_SO_4_, pH 7.0] and spread onto Wx agar plates containing 5 mM PDC as the sole carbon and energy source. Colonies with distinct morphological characteristics were selected from each sample, yielding two to four isolates per sample and a total of 82 PDC-assimilating strains.

### Evaluation of inducible PDC conversion activity

The isolated PDC-assimilating strains and *Comamonas* sp. E6 were cultured for 24 h in LB medium (non-inducing conditions) or LB medium containing 10 mM PDC (inducing conditions). Cells were harvested by centrifugation at 4800 × *g* for 5 min at 4 °C, washed twice with Wx buffer, and resuspended in the same buffer to prepare resting cells. Resting cells were adjusted to an OD_600_ of 0.1 or 0.2 and incubated with 500 μM PDC in a 96-well plate at 30 °C with shaking at 567 cycles per minute (cpm) using an EPOCH2 microplate reader (Agilent BioTek Inc.). The absorbance at 310 nm, corresponding to the absorption maximum of PDC, was monitored over time. The time required for the absorbance at 310 nm to reach a stable minimum, reflecting complete PDC consumption, was defined as the complete PDC conversion time. Inducibility was assessed by comparing PDC conversion times under non-inducing and inducing conditions.

For strains showing inducibility, resting cells prepared after cultivation in LB with or without 10 mM PDC were adjusted to an OD_600_ of 1.0 and incubated with 500 µM PDC at 30 °C. PDC concentrations at the start of the reaction and after 1 h were determined by high-performance liquid chromatography (HPLC), and the PDC conversion rate (µM PDC·h^−1^·OD_600_^−1^) was calculated.

### HPLC analysis

All HPLC analyses were performed on an ACQUITY iClass UPLC system (Waters). Samples were filtered through a 0.2 µm PTFE membrane filter (Captiva Econofilter, Agilent), and separated on a TSKgel ODS-140HPT column (2.3 µm, 2.1 × 100 mm; Tosoh) at a flow rate of 0.5 mL/min. For routine analyses, the mobile phase consisted of a solvent A (acetonitrile containing 0.1% formic acid) and solvent B (water containing 0.1% formic acid), with the following gradient: 0–3.0 min, 12–60% A; 3.0–3.1 min, 60–12% A; 3.1–4.0 min, 12% A. For the analysis of PDC produced from black liquor extract, solvent A (acetonitrile containing 0.5% phosphoric acid) and solvent B (water containing 0.5% phosphoric acid) were used with the following gradient: 0–4.0 min, 1–10% A; 4.0–5.0 min, 10–100% A; 5.0–6.0 min, 100% A; 6.0–6.1 min, 100–1% A; 6.1–7.0 min, 1% A. PDC and vanillic acid were detected at 315 nm and 260 nm, respectively. The aromatic components in the black liquor extract were analyzed as previously described [14].

### 16S rRNA gene sequencing and phylogenetic analysis

The 16S rRNA gene was amplified by PCR using primers 533f and 1525r with total DNA extracted from each strain as the template. The amplified products were sequenced by Eurofins Genomics (Japan). Homology searches of the 16S rRNA gene sequences were performed using NCBI BLASTn against the 16S ribosomal RNA sequences (Bacteria and Archaea) database [30]. Pairwise alignments were carried out using EMBOSS Needle [31], and phylogenetic trees were constructed by the maximum likelihood method using MEGA11 [32].

### Whole-genome sequencing and identification of putative PDC hydrolase genes

Whole-genome sequencing of total DNA extracted from each strain was performed on an Illumina NovaSeq 6000 platform by Novogene (China). The obtained reads were assembled using VelvetOptimiser v2.2.6 [33] and annotated using DFAST [34]. The draft genome sequences were deposited in GenBank under the following accession numbers: *Cupriavidus* sp. 8B, NZ_BAAILO000000000.1; *Burkholderia* sp. 3C, NZ_BAAILN000000000.1; *Paraburkholderia* sp. 2C, NZ_BAAILL000000000.1; *Pseudomonas* sp. 3A, NZ_BAAILM000000000.1; and *Novosphingobium* sp. 11B, NZ_BAAILP000000000.1. Putative PDC hydrolase genes were identified by BLASTp searches of the predicted protein sequences from each genome using the amino acid sequences of known PDC hydrolases listed in Table S3.

### Construction of reporter plasmids

Primers used for plasmid construction are listed in Table S2, and the sequences of synthetic genes are provided in Table S4. A synthetic gene consisting of a Shine–Dalgarno (SD) sequence followed by an mNeonGreen coding sequence codon-optimized for SYK-6 was cloned into the SmaI site of pSEVA231 using NEBuilder HiFi DNA Assembly Master Mix (New England Biolabs) to generate pPNS. Promoter regions (400–420 bp upstream of putative PDC hydrolase genes) were amplified by PCR using total DNA from each strain as the template. Transcriptional regulator genes were codon-optimized for SYK-6 and synthesized as gene fragments preceded by the *bla* promoter (P_*amp*_) and an SD sequence. The promoter regions and transcriptional regulator genes were then cloned into the EcoRV site of pPNS to construct candidate biosensor plasmids. Gene synthesis was outsourced to Twist Bioscience (USA).

### Construction of reporter strains and fluorescence assays

Each reporter plasmid was introduced into the SYK-6 Δ*ligI* or Δ*ligAB* strains by triparental mating to generate reporter strains. Δ*ligI* was used as the host for all reporter strains, whereas Δ*ligAB* was used to evaluate the specificity of PdcR. Each reporter strain was precultured in Wx-SEMP medium for 24 h, harvested by centrifugation at 4800 × *g* for 5 min at 4 °C, washed twice with Wx buffer, and resuspended in the same buffer.

To evaluate substrate responsiveness, washed cells were inoculated into 200 µL of Wx-SEMP medium containing 0–50 mM PDC, 0–5 mM vanillic acid, or PCA, or 0.25–1.00% (v/v) black liquor extract in 96-well plates to an OD_600_ of 0.2. The cultures were incubated at 30 °C with shaking at 567 cpm in a Synergy H1 microplate reader (Agilent BioTek Inc.), and OD_600_ and mNeonGreen fluorescence (excitation, 506 nm; emission, 547 nm) were measured over time. Fluorescence intensity was expressed as relative fluorescence units (RFU) and normalized to cell density (OD_600_), yielding RFU/OD_600_.

For evaluation of the time lag between PDC production and fluorescence, washed cells of Δ*ligI*(pCup1) were inoculated into 10 mL of Wx-SEMP medium containing 1 or 5 mM vanillic acid in L-shaped tubes to an OD_600_ of 0.2 and incubated at 30 °C with shaking at 160 rpm. At multiple time points up to 40 h, OD_600_ and fluorescence (RFU) were measured using a microplate reader, and culture supernatants were collected in parallel for determination of vanillic acid and PDC concentrations by HPLC.

To evaluate the response to black liquor extract, washed cells of Δ*ligI*(pCup1) were inoculated into 200 µL of Wx-SEMP medium containing 0.25–1.00% (v/v) black liquor extract in 96-well plates to an OD_600_ of 0.2 and incubated at 30 °C with shaking at 567 cpm in a microplate reader. Because the color of the black liquor extract precluded accurate OD_600_ measurements, fluorescence (RFU) alone was used as the indicator of PDC production. Culture supernatants were collected when RFU reached a plateau, and PDC concentrations were quantified by HPLC. The composition of major lignin-derived aromatic compounds in the original black liquor before extraction is shown in Table S5.

## Results

### Isolation of PDC-assimilating bacteria with inducible PDC-converting activity

**Fig. 2:**
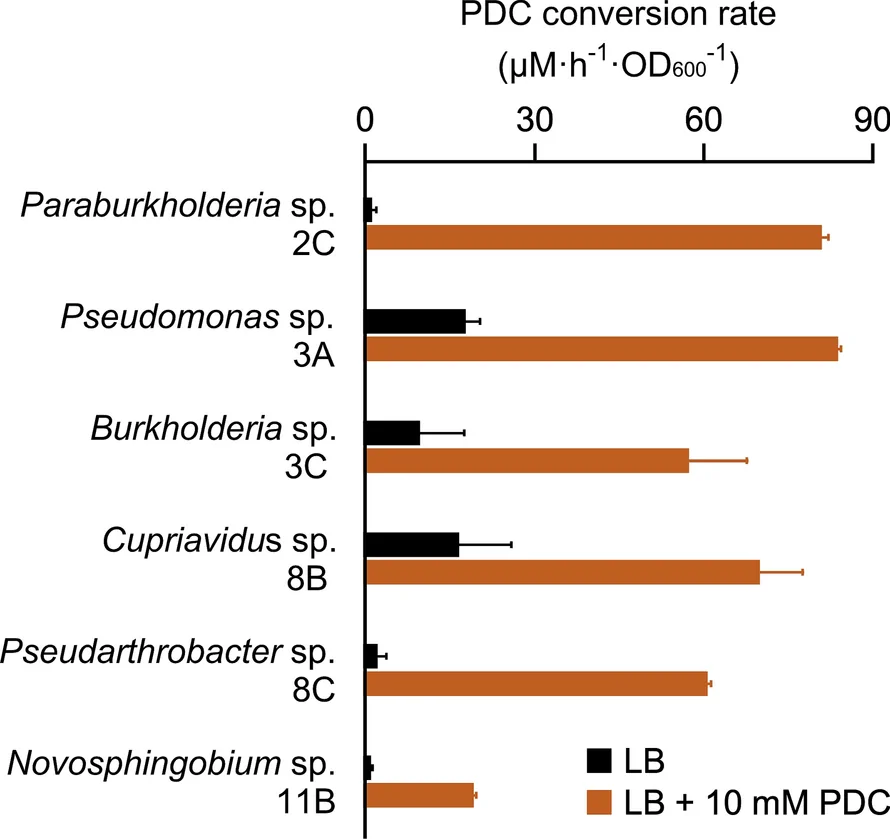
Screening of bacteria exhibiting inducible PDC conversion activity. Strains showing significant induction in the preliminary screen (Fig. S1) were cultured in LB (non-inducing conditions, black) or LB containing 10 mM PDC (inducing conditions, orange), and PDC conversion rates were determined using resting cells. Each value represents the mean ± standard error from three independent experiments

To identify PDC-responsive transcriptional regulatory systems, we hypothesized that bacteria capable of assimilating PDC as a sole carbon source would harbor such regulatory systems and accordingly screened environmental samples for such bacteria. A total of 82 PDC-assimilating strains were isolated from environmental samples collected at 26 locations, including decayed wood and forest soils. Among these isolates, 16 strains exhibited induction of PDC-converting activity upon cultivation in the presence of PDC (Fig. S1). Six strains showed particularly strong induction, with 4–81-fold increases in PDC conversion rates under inducing conditions (Fig. 2), and were selected as candidates harboring PDC-responsive transcriptional regulatory systems. Based on 16S rRNA gene sequence analysis, these strains were classified into the genera *Cupriavidus*, *Burkholderia*, and *Paraburkholderia* (β-Proteobacteria); *Pseudomonas* (γ-Proteobacteria); *Novosphingobium* (α-Proteobacteria); and *Pseudarthrobacter* (Gram-positive bacteria) (Fig. S2, Table S6). For subsequent analyses, we focused on the Gram-negative strains: *Cupriavidus* sp. 8B, *Burkholderia* sp. 3C, *Paraburkholderia* sp. 2C, *Pseudomonas* sp. 3A, and *Novosphingobium* sp. 11B, considering that the transcriptional regulatory systems would be evaluated in the α-proteobacterial host strain SYK-6.

### Identification of PDC-responsive transcriptional regulatory systems through genome analysis

**Fig. 3:**
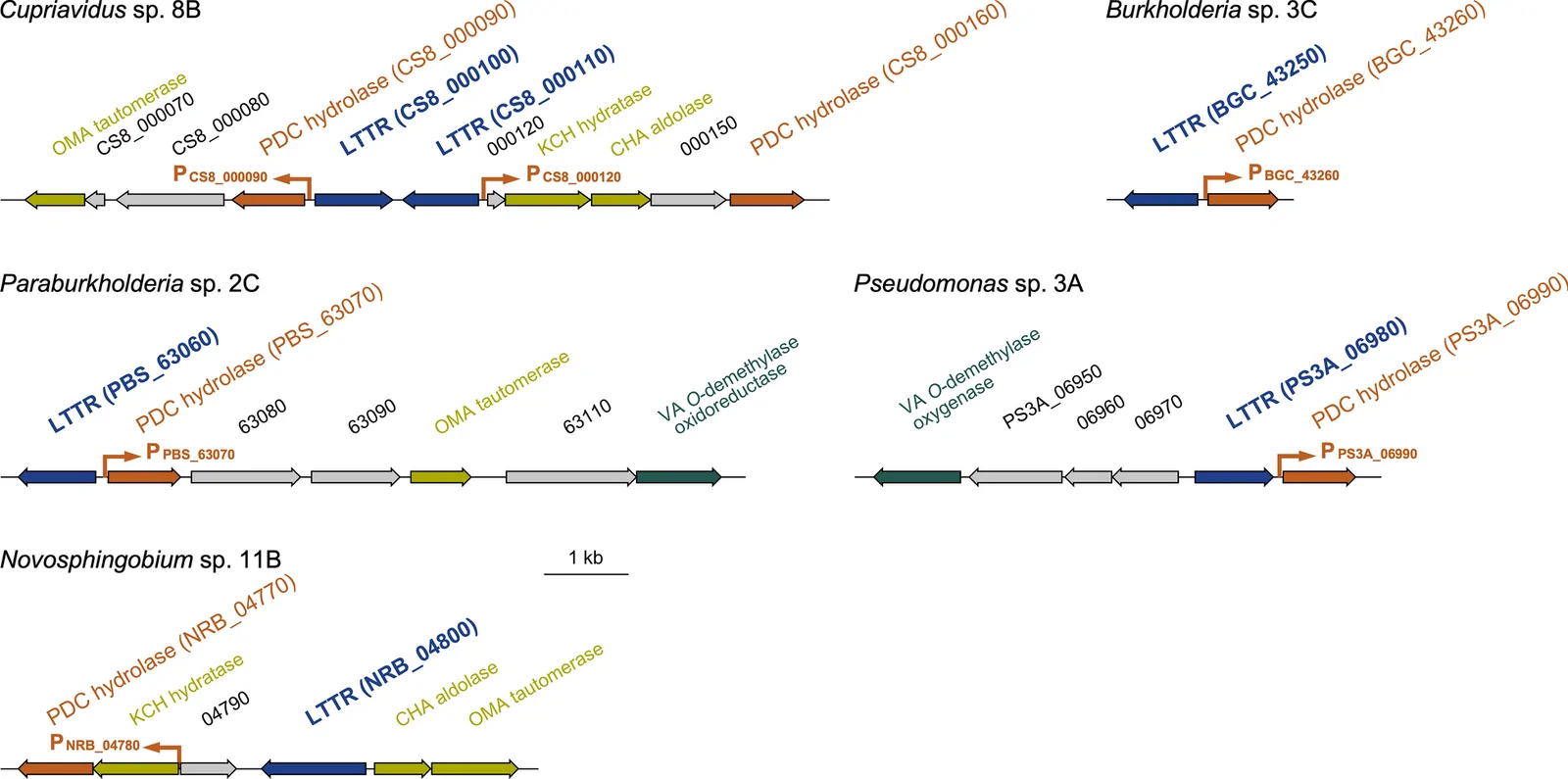
Gene organization around putative PDC hydrolase genes. Open reading frames (ORFs) in regions surrounding the putative PDC hydrolase genes are shown. Color coding: orange, putative PDC hydrolase genes; yellow, genes encoding enzymes acting downstream of PDC in the PCA 4,5-cleavage pathway; green, genes involved in the metabolism of aromatic compounds potentially converted via PDC; blue, genes encoding putative LysR-type transcriptional regulators selected as candidates; gray, genes of unknown or unrelated function. Promoters of putative PDC hydrolase genes predicted to be induced by PDC are indicated by orange bent arrows. Abbreviations: PDC, 2-pyrone-4,6-dicarboxylic acid; OMA, 4-oxalomesaconic acid; KCH, 2-keto-4-carboxy-3-hexenedioic acid; CHA, 4-carboxy-4-hydroxy-2-oxoadipic acid; VA, vanillic acid; LTTR, LysR-type transcriptional regulator

The genomes of the five selected strains were sequenced and analyzed (Table S7). To identify promoters potentially inducible by PDC, we searched each genome for genes homologous to known PDC hydrolases. One or two putative PDC hydrolase genes with more than 40% amino acid sequence identity to known PDC hydrolases were found in each genome (Table S3). Genes encoding enzymes of the PCA 4,5-cleavage pathway were found near the putative PDC hydrolase genes in *Cupriavidus* sp. 8B and *Novosphingobium* sp. 11B, whereas genes encoding vanillic acid *O*-demethylase were located near the corresponding genes in *Paraburkholderia* sp. 2C and *Pseudomonas* sp. 3A (Fig. 3, Table S8). In contrast, no genes encoding metabolic enzymes potentially involved in PDC metabolism were found in the vicinity of the putative PDC hydrolase gene in *Burkholderia* sp. 3C. Notably, two putative PDC hydrolase genes (CS8_000090 and CS8_000160) were located in close proximity in *Cupriavidus* sp. 8B.

Candidate PDC-responsive transcriptional regulator genes and their cognate promoter regions were then selected from regions flanking the putative PDC hydrolase genes. In *Cupriavidus* sp. 8B (CS8_000090), *Burkholderia* sp. 3C, *Paraburkholderia* sp. 2C, and *Pseudomonas* sp. 3A, the putative PDC hydrolase genes were preceded by genes encoding putative LysR-type transcriptional regulators (LTTRs). Accordingly, the upstream regions of these PDC hydrolase genes were selected as candidate promoter regions (Fig. 3, Table S4). For CS8_000160 in *Cupriavidus* sp. 8B and NRB_04770 in *Novosphingobium* sp. 11B, genes predicted to form operons were located upstream of the PDC hydrolase genes; therefore, the upstream regions of CS8_000120 and NRB_04780 were selected as the candidate promoter regions. LTTR genes located near each putative PDC hydrolase gene were selected as candidate regulatory genes (Fig. 3).

### Evaluation of the PDC responsiveness of candidate transcriptional regulatory systems

**Fig. 4:**
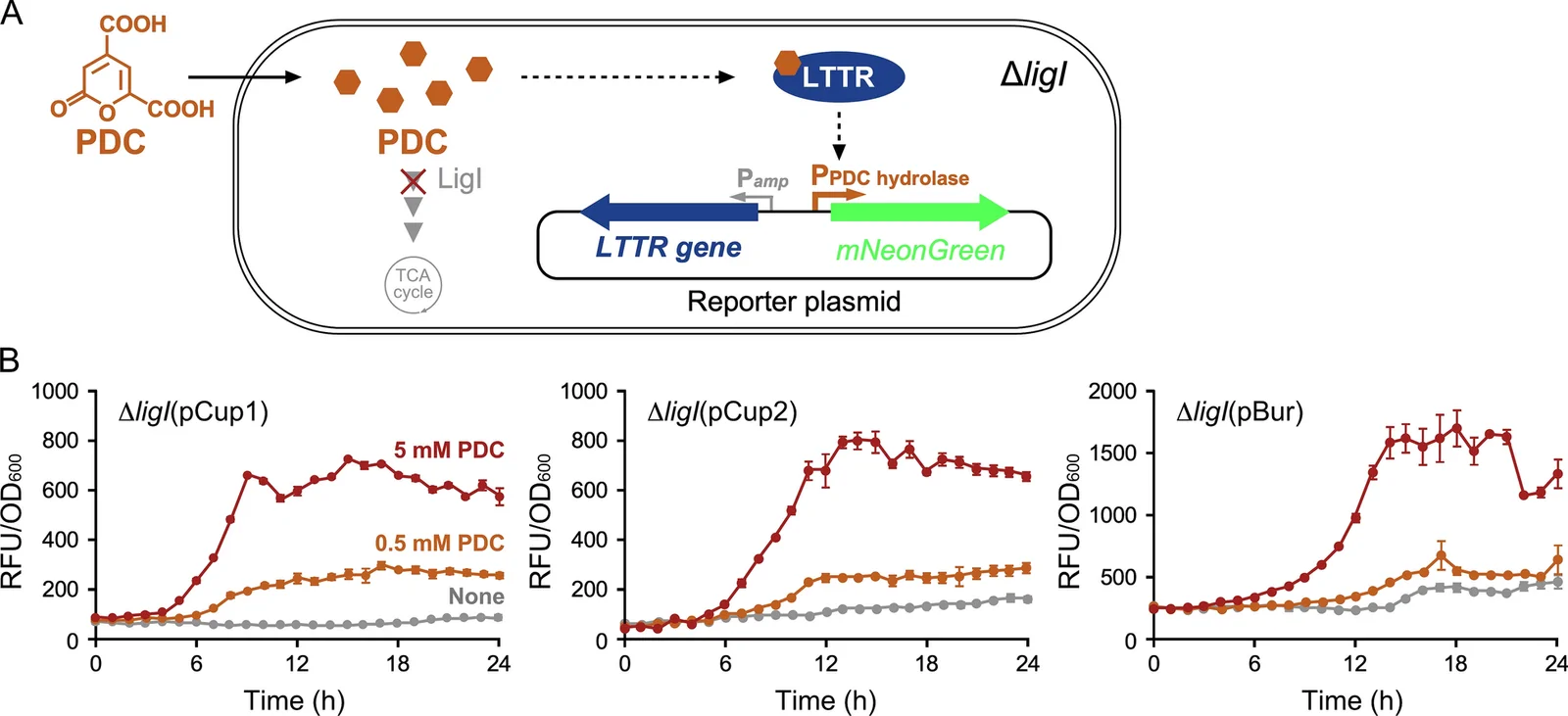
Evaluation of candidate PDC biosensors using a reporter assay. **A** Schematic representation of the reporter system constructed in Δ*ligI*. PDC accumulated in Δ*ligI* is detected by the LTTR encoded on the reporter plasmid, which controls *mNeonGreen* expression via the PDC hydrolase gene promoter (P_PDC hydrolase_). The P_PDC hydrolase_ and SD sequences are shown in Table S4. P_*amp*_, *bla* promoter. **B** Evaluation of PDC responsiveness. Δ*ligI* harboring pCup1, pCup2, or pBur was cultured in Wx-SEMP medium with or without 0.5 or 5 mM PDC, and RFU/OD_600_ was measured over time. Each value represents the mean ± standard error from three independent experiments. Results for the vector control and other candidate PDC biosensor plasmids are shown in Fig. S3

**Fig. 5:**
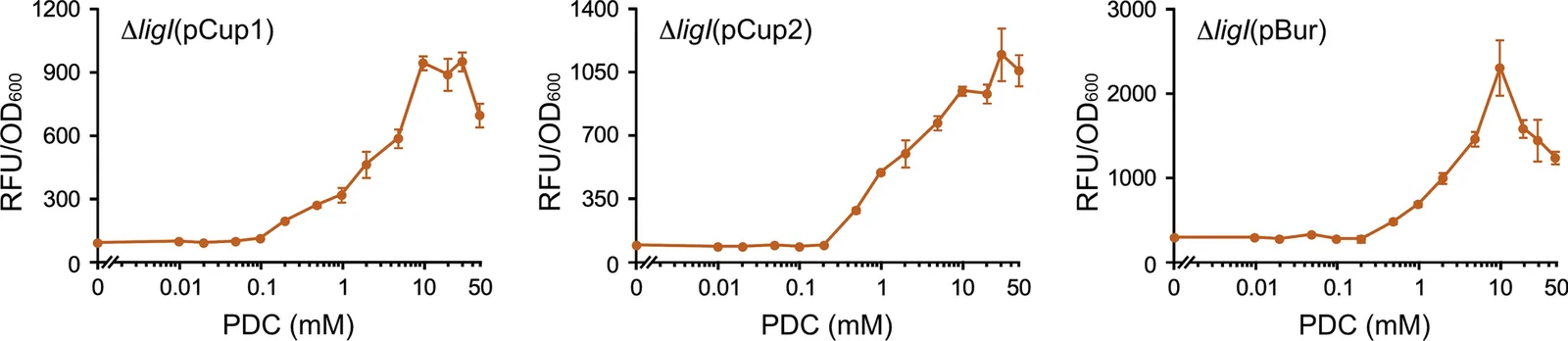
Evaluation of responsiveness to PDC. Δ*ligI*(pCup1), Δ*ligI*(pCup2), and Δ*ligI*(pBur) were cultured in Wx-SEMP medium with or without 0.01–50 mM PDC, and RFU/OD_600_ was measured over time. RFU/OD_600_ at the time point when both RFU and OD_600_ reached plateaus is shown. Each value represents the mean ± standard error from three independent experiments

Each selected LTTR gene and its cognate promoter region were cloned into the pPNS vector carrying *mNeonGreen* as a reporter gene to construct reporter plasmids (Table S4). Each reporter plasmid was introduced into Δ*ligI*, a *ligI*-deficient PDC-accumulating mutant of SYK-6, to generate candidate biosensor strains (Fig. 4A). When cultivated in Wx-SEMP medium under non-inducing conditions or in the presence of 0.5 or 5 mM PDC, Δ*ligI* strains harboring the reporter plasmids pCup1 or pCup2 from *Cupriavidus* sp. 8B or pBur from *Burkholderia* sp. 3C exhibited marked, PDC concentration-dependent increases in fluorescence intensity (Figs. 4B, S3). The PDC concentration ranges eliciting fluorescence responses were then examined for Δ*ligI*(pCup1), Δ*ligI*(pCup2), and Δ*ligI*(pBur) (Fig. 5). For all three biosensors, the fluorescence response increased in a PDC concentration-dependent manner and reached its maximum or a plateau around 10 mM PDC. Defining the dynamic range as the concentration range over which the fluorescence response increased in a concentration-dependent manner, the dynamic ranges of Δ*ligI*(pCup1), Δ*ligI*(pCup2), and Δ*ligI*(pBur) were 0.2–10 mM, 0.5–10 mM, and 0.5–10 mM PDC, respectively. Among these, Δ*ligI*(pCup1) exhibited the widest dynamic range for PDC detection under the assay conditions used in this study. Therefore, Δ*ligI*(pCup1) was selected for subsequent analyses, and the LTTR and its cognate promoter of this PDC-responsive transcriptional regulatory system were designated PdcR and P_PDC_, respectively.

### Response range and specificity of the PDC biosensor

**Fig. 6:**
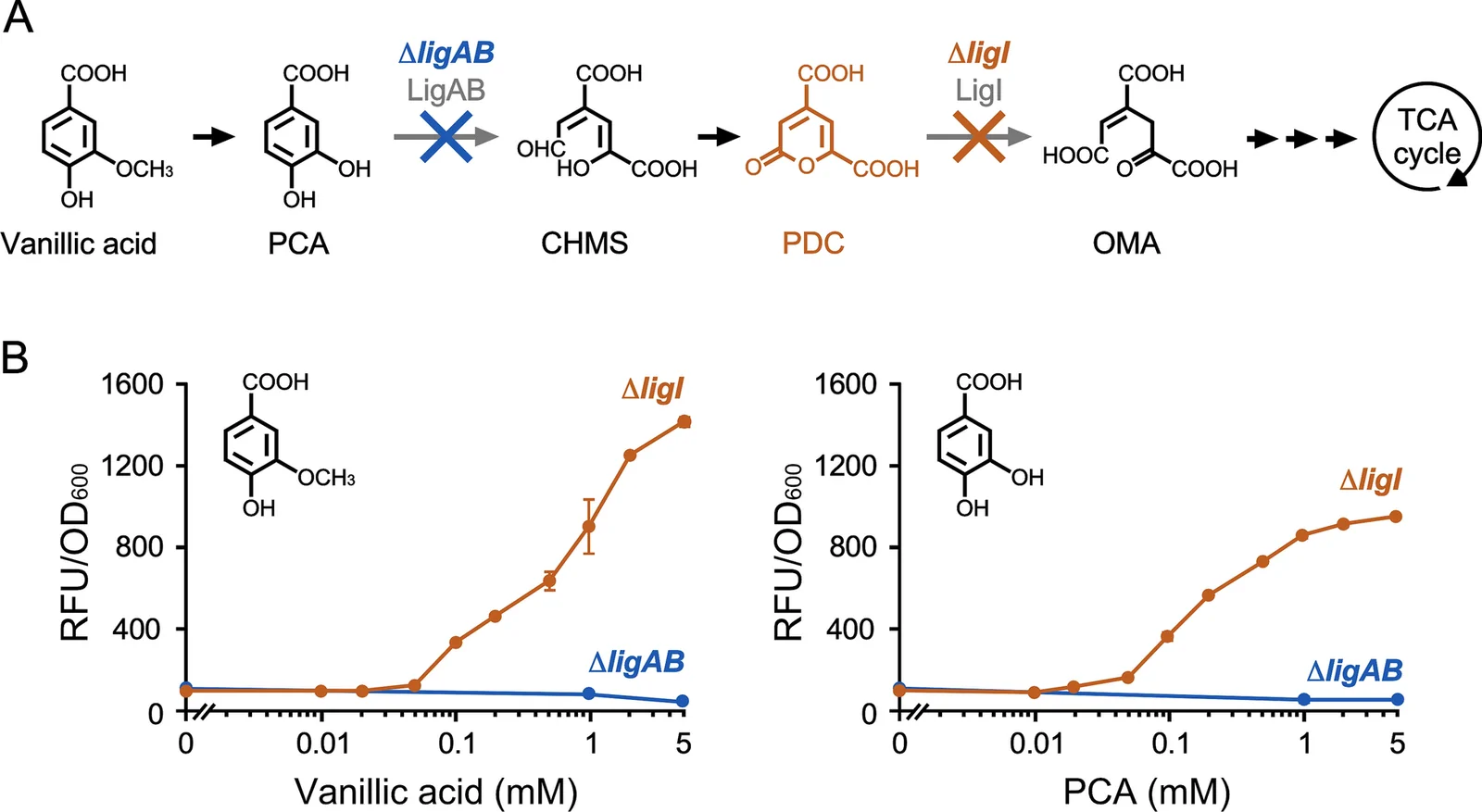
PDC specificity of the biosensor. **A** Catabolic pathway of vanillic acid in SYK-6. Δ*ligAB* and Δ*ligI* are deficient in the conversion of PCA and PDC, respectively. Abbreviations: CHMS, 4-carboxy-2-hydroxymuconate-6-semialdehyde; OMA, 4-oxalomesaconic acid. **B** Specificity of PdcR for PDC confirmed using vanillic acid and PCA as substrates. Δ*ligI*(pCup1) was cultured in Wx-SEMP medium with or without 0.01–5 mM vanillic acid or PCA. Δ*ligAB*(pCup1) was cultured in the same medium with or without 1 or 5 mM vanillic acid or PCA. RFU/OD_600_ at the time point when both RFU and OD_600_ reached plateaus is shown. Each value represents the mean ± standard error from three independent experiments

The response of Δ*ligI*(pCup1) was evaluated using vanillic acid and PCA as substrates, both of which are metabolized to PDC in SYK-6 (Fig. 6A). For both substrates, a concentration-dependent increase in fluorescence intensity was observed over the range of 0.1–5 mM added substrate, indicating that Δ*ligI*(pCup1) responds to PDC produced from lignin-derived aromatic compounds (Fig. 6B). To exclude the possibility that endogenous regulators of SYK-6 act on P_PDC_, a control plasmid (pCup0) containing only P_PDC_ without *pdcR* was constructed and introduced into Δ*ligI*. No fluorescence induction was observed in Δ*ligI*(pCup0) even in the presence of PDC (Fig. S4), confirming that the PDC response in Δ*ligI*(pCup1) depends on PdcR. Notably, Δ*ligI*(pCup0) exhibited higher basal fluorescence in the absence of PDC but lower fluorescence in its presence compared with Δ*ligI*(pCup1) (Figs. 4, S4), suggesting that PdcR functions as both a repressor and an activator of transcription from P_PDC_. To further evaluate the specificity of PdcR, Δ*ligAB*(pCup1), a PCA catabolism-deficient mutant carrying pCup1, was cultivated in the presence of vanillic acid or PCA. No increase in fluorescence intensity was observed under either condition, indicating that PdcR responds specifically to PDC rather than to its upstream metabolites (Fig. 6B).

### Real-time detection and responsiveness to biomass-derived mixtures

One potential application of the PDC biosensor is the screening for SYK-6 mutant strains with improved conversion efficiency of lignin-derived aromatic compounds to PDC. For this application, a fluorescence response with minimal lag relative to PDC production is desirable. To assess this, the correlation between PDC production from vanillic acid and the fluorescence responses (RFU and RFU/OD_600_) was evaluated using Δ*ligI*(pCup1). The RFU increased synchronously with PDC concentration and reached a plateau when PDC concentration became maximal (Fig. S5). In contrast, RFU/OD_600_ tended to plateau earlier in the presence of 5 mM vanillic acid despite continued PDC production (Fig. S5). These results suggest that, in this time-course experiment, RFU may serve as an indicator for real-time evaluation of PDC production from lignin-derived aromatic compounds.

**Fig. 7:**
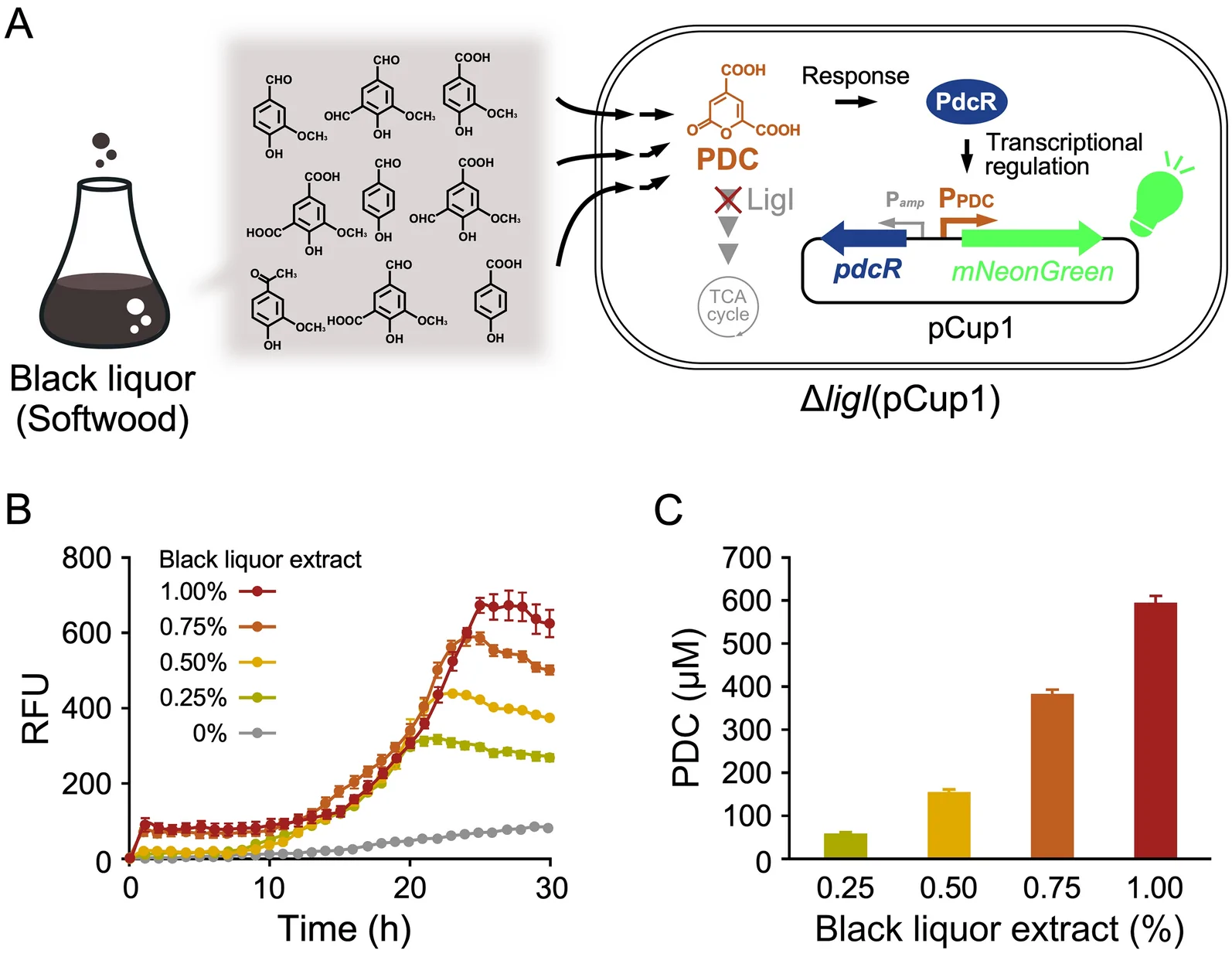
Responsiveness of Δ*ligI*(pCup1) to softwood-derived black liquor extract. **A** Schematic representation of PDC detection from softwood-derived black liquor extract. Δ*ligI*(pCup1) converts aromatic compounds in the black liquor extract to PDC and detects it as a fluorescence signal. **B** Fluorescence response to black liquor extract. Δ*ligI*(pCup1) was cultured in Wx-SEMP medium with or without 0.25–1.00% (v/v) softwood-derived black liquor extract, and RFU was measured over time. Because the black liquor extract exhibited background fluorescence, RFU values were corrected by subtracting the initial RFU at time zero. Each value represents the mean ± standard error from three independent experiments. **C** PDC concentrations at the RFU plateau. Culture supernatants were collected when RFU reached a plateau, and PDC concentrations were quantified by HPLC. Each value represents the mean ± standard error from three independent experiments

To simulate PDC production from lignocellulosic biomass, Δ*ligI*(pCup1) was cultivated in Wx-SEMP medium containing 0.25–1.00% (v/v) black liquor extract prepared from softwood by oxygen-soda anthraquinone pulping, a complex mixture of lignin-derived aromatic compounds (Fig. 7A). The major aromatic components of the extract were vanillin, vanillic acid, acetovanillone, and 5-carboxyvanillin, with concentrations of 40.1, 11.4, 3.91, and 3.17 mM, respectively. Because the coloration of the extract interfered with accurate OD_600_ measurement, RFU rather than RFU/OD_600_ was used as the indicator of the fluorescence response. Both the maximum RFU (Fig. 7B) and the PDC concentration at the RFU plateau (Fig. 7C) increased with extract concentration, indicating that Δ*ligI*(pCup1) responds to PDC produced from lignin-derived aromatic mixture in the extract and that RFU can be used to estimate PDC production under these conditions.

## Discussion

The PDC biosensor Δ*ligI*(pCup1) constructed in this study responded not only to exogenously supplied PDC but also to PDC generated intracellularly through the conversion of lignin-derived aromatic compounds (Figs. 5, 6). When vanillic acid or PCA was used as a substrate, a concentration-dependent increase in fluorescence intensity was observed over the range of 0.1–5 mM, indicating that PDC production from these substrates can be quantitatively monitored (Fig. 6). No fluorescence induction was observed in response to the upstream metabolites of PDC, suggesting that PdcR responds specifically to PDC rather than to its upstream metabolites. Monitoring RFU enabled real-time evaluation of PDC production from lignin-derived aromatic compounds. Notably, a concentration-dependent fluorescence response was also observed for PDC produced from softwood black liquor extract (Fig. 7). The two additional PDC-responsive systems identified in this study, pCup2 and pBur, could expand the available repertoire of PDC-responsive biosensors (Fig. 5). Their detailed characterization in future studies may reveal distinct properties suitable for specific screening applications.

Δ*ligI*(pCup1) has practical significance not only as a detection tool but also as a screening platform for strains with improved PDC production. To date, evaluation of PDC production from lignin-derived aromatic compounds has largely relied on HPLC analysis [8–14, 16–18], making large-scale screening of mutant or evolved strains impractical. In contrast, fluorescence-based biosensors enable simultaneous evaluation of multiple samples using a microplate reader or a droplet microfluidics platform, thereby overcoming throughput limitations [21–23]. Furthermore, the use of SYK-6 as the host integrates the detection system and production host into a single strain, simplifying implementation of the screening platform [14–18]. The fluorescence response associated with PDC production from lignocellulosic biomass-derived black liquor extract further demonstrates that this biosensor functions effectively for lignin valorization. Beyond the oxygen-soda anthraquinone pulping in this study, vanillin, vanillic acid, and other guaiacyl-type aromatic compounds are commonly generated during various oxidative depolymerization processes of softwood lignin, such as tetrabutylammonium hydroxide-mediated aerobic oxidation [35, 36] and membrane reactor-based oxidative depolymerization [37]. In addition, metabolically engineered SYK-6 can efficiently convert syringyl-type compounds, such as syringic acid, which are abundant in oxidative depolymerization products of hardwood lignin, into PDC [14]. Therefore, aromatic compounds released from different types of lignin by oxidative depolymerization can serve as substrates for PDC production in SYK-6-based strains. Taken together, engineered SYK-6-based PDC biosensors are broadly applicable to screening for PDC production from oxidative depolymerization products of various lignin sources. Furthermore, the PdcR–P_PDC_ system identified here is expected to be applicable beyond SYK-6: introduction into previously engineered PDC-producing strains of *Pseudomonas putida* [8–11] and *Novosphingobium aromaticivorans* [12, 13] would provide a direct route toward accelerated strain improvement in these chassis.

The PDC biosensor can be combined with diverse strain improvement strategies, including random mutagenesis, adaptive laboratory evolution, and metagenomics-based enzyme discovery [38–43]. For example, combining adaptive laboratory evolution with a product-responsive biosensor has been shown to simultaneously improve host tolerance and production titer, as demonstrated in a PCA production system [42]. Applied to lignin valorization, screening with the PDC biosensor would enable selection of strains with improved PDC production rates based on real-time fluorescence monitoring. In addition, combining the PDC biosensor with metagenomic libraries represents a particularly promising application [44, 45]. Even in SYK-6, which catabolizes a wide range of aromatic compounds, the enzymes responsible for converting aromatic oligomers, which constitute a major fraction of depolymerized lignin [46], remain unidentified. The genomes of uncultured microorganisms from diverse environments are expected to harbor novel enzymes involved in the degradation of these oligomers. By using Δ*ligI*(pCup1) harboring a metagenomic library, fluorescence signals would be generated only when metagenome-derived enzymes convert aromatic oligomers into compounds that can be catabolized through the SYK-6 pathway to PDC, enabling direct identification of novel enzyme genes involved in PDC production from aromatic oligomers. In practice, this biosensor can support at least two screening criteria. For selecting strains that produce PDC at higher concentrations, the maximum fluorescence reflects the amount of PDC produced, since a higher concentration of PDC generated from upstream substrates was accompanied by a higher fluorescence at the RFU plateau (Figs. 7, S5). For selecting strains that produce PDC more rapidly, the time course of fluorescence reflects the rate of PDC production, since the fluorescence increased in synchrony with the concentration of PDC generated from vanillic acid in the time-course assay (Fig. S5). The biosensor thus provides a practical readout for ranking strains by either the amount or the rate of PDC production from lignin-derived aromatic compounds. Several considerations nevertheless remain. Unlike such relative comparisons, estimating the absolute concentration of PDC produced from the fluorescence values requires caution. The process leading to the fluorescence response differs between exogenously supplied PDC and PDC generated intracellularly from upstream substrates. Although the two responses are unlikely to differ markedly, the relationship between fluorescence level and PDC concentration established with exogenous PDC cannot necessarily be applied directly to the quantification of PDC produced from upstream substrates. Quantitative estimation of the produced PDC concentration would therefore require, for each substrate, constructing a calibration curve that relates the fluorescence output to the PDC concentration based on time-course measurements during substrate conversion. Furthermore, signal normalization remains an important challenge for practical screening using colored lignin-derived substrates such as black liquor extract. In the experiments with black liquor extract in this study, the coloration of the extract precluded accurate cell density measurement, and the fluorescence signal could not be normalized to OD_600_. For future applications, normalization independent of OD_600,_ using a constitutively expressed fluorescent protein with an emission wavelength distinct from that of the reporter (such as mCherry) to correct the reporter fluorescence, would provide an effective solution.

Transcriptional regulator-based biosensors are versatile tools for measuring target ligand concentrations, with applications spanning metabolic engineering, environmental analysis, and medical diagnostics [47–49]. However, their construction relies on the availability of transcriptional regulators responsive to target compounds, and expanding the repertoire of regulators that recognize new ligands remains a key challenge [50]. Engineering approaches such as computational design and directed evolution have been developed to address this challenge [51]; however, these methods are constrained by the structures of existing regulators and thus limited in the range of ligands that the engineered variants can recognize. Although biosensors for several lignin-derived compounds such as vanillin [44, 52], vanillic acid [53], and muconic acid [54] have been developed using existing transcriptional regulators from well-characterized aromatic degradation pathways [24], no PDC-responsive regulatory system had been identified prior to this study. The wet-lab approach described here therefore provides a practical route to construct biosensors targeting compounds for which no natural regulatory system is known. Here, we employed a traditional wet-lab approach to identify a PDC-responsive transcriptional regulator: screening of PDC-assimilating bacteria, selection of strains exhibiting inducible PDC-converting activity, genome-based identification of regulator genes and promoters located proximal to conversion enzyme genes, and reporter-based evaluation of PDC responsiveness. This approach is effective for identifying novel natural transcriptional regulatory systems responsive to specific compounds without relying on sequence information from known regulators.

Genome analysis of PDC-assimilating bacteria revealed that genes predicted to encode dicarboxylate transporters were conserved near putative PDC hydrolase genes (Table S8). Homologs of these transporter genes were also identified in SYK-6, with amino acid sequence identities of 45.0–80.8% (Table S9), suggesting that these transporters are involved in PDC uptake by both PDC-assimilating bacteria and the biosensor host SYK-6.

Compared with Δ*ligI*(pCup1), which retains *pdcR*, Δ*ligI*(pCup0), lacking *pdcR*, exhibited higher basal fluorescence in the absence of PDC but lower fluorescence in its presence (Figs. 4, S4), suggesting that PdcR functions as both a repressor and an activator of transcription from P_PDC_. This dual regulatory mode is consistent with the sliding dimer mechanism characteristic of LTTRs, to which PdcR belongs [55, 56]. Characterization of the PdcR binding sequence and its positional relationship with P_PDC_ would provide a foundation for sequence optimization to improve biosensor sensitivity and dynamic range. In the assays using exogenously supplied PDC, the biosensors reached the upper limit of their response around 10 mM (Fig. 5). If a broader response range is required for future applications, the dynamic range and sensitivity of the PdcR-based PDC biosensor could potentially be tuned by sensor engineering. Indeed, in LTTR-based biosensors, improvements in dynamic range and sensitivity have been achieved through engineering of transcriptional regulator variants and of operator and promoter sequences [49, 57, 58].

## Conclusions

In this study, we developed a PDC-responsive biosensor using *S. lignivorans* SYK-6 as the host to enable high-throughput screening for strains with improved PDC production. Through combining the screening of PDC-assimilating bacteria followed by genome analysis, we identified the PdcR–P_PDC_ transcriptional regulatory system from *Cupriavidus* sp. 8B and constructed the PDC-responsive biosensor Δ*ligI*(pCup1). This biosensor would enable real-time fluorescence-based detection of PDC production from lignocellulosic biomass-derived black liquor extract and is applicable to large-scale screening under a wide range of lignin valorization conditions. By integrating this biosensor with diverse strain engineering approaches, including random mutagenesis, adaptive laboratory evolution, and metagenomics-based enzyme discovery, the Δ*ligI*(pCup1) system would provide a platform for efficient selection of SYK-6 strains with enhanced PDC production capability.

## Supplementary Information

Additional File 1: Table S1. Strains and plasmids used in this study. Table S2. Nucleotide sequences of primers used in this study. Table S3. PDC hydrolase genes identified in each strain. Table S4. Construction of reporter plasmids and sequences of synthetic genes. Table S5. Monomer composition of softwood black liquor. Table S6. Taxonomic classification of the candidate strains. Table S7. Summary of genome assembly statistics for the analyzed strains. Table S8. Genes identified in the vicinity of putative PDC hydrolase genes. Table S9. Homology analysis of dicarboxylate symporters. Fig. S1. Inducibility of PDC conversion activity in isolated bacteria. Fig. S2. Phylogenetic analysis of bacteria exhibiting inducible PDC conversion activity based on 16S rRNA gene sequences. Fig. S3. Evaluation of PDC responsiveness. Fig. S4. Evaluation of PDC responsiveness in Δ*ligI*(pCup0). Fig. S5. Evaluation of the timing of PDC production and fluorescence response.

## Data Availability

The draft genome sequences generated and analyzed in this study have been deposited in GenBank under the following accession numbers: *Cupriavidus* sp. 8B, NZ_BAAILO000000000.1; *Burkholderia* sp. 3 C, NZ_BAAILN000000000.1; *Paraburkholderia* sp. 2 C, NZ_BAAILL000000000.1; *Pseudomonas* sp. 3 A, NZ_BAAILM000000000.1; and *Novosphingobium* sp. 11B, NZ_BAAILP000000000.1. All other datasets supporting the conclusions of this article are included within the article and its additional file.

## Abbreviations

HPLC: High-performance liquid chromatography
LB: Lysogeny Broth
LTTR: LysR-type transcriptional regulator
PCA: Protocatechuic acid
PDC: 2-Pyrone-4,6-dicarboxylic acid
RFU: Relative fluorescence units
SD: Shine–Dalgarno

## References

[1] Ragauskas AJ, Beckham GT, Biddy MJ, Chandra R, Chen F, Davis MF, et al. Lignin valorization: improving lignin processing in the biorefinery. Science. 2014;344(6185):1246843. 10.1126/science.1246843 24833396

[2] Zakzeski J, Bruijnincx PCA, Jongerius AL, Weckhuysen BM. The catalytic valorization of lignin for the production of renewable chemicals. Chem Rev. 2010;110(6):3552–99. 10.1021/cr900354u 20218547

[3] Linger JG, Vardon DR, Guarnieri MT, Karp EM, Hunsinger GB, Franden MA, et al. Lignin valorization through integrated biological funneling and chemical catalysis. Proc Natl Acad Sci U S A. 2014;111(33):12013–8. 10.1073/pnas.1410657111 PMC4143016 25092344

[4] Weiland F, Kohlstedt M, Wittmann C. Guiding stars to the field of dreams: metabolically engineered pathways and microbial platforms for a sustainable lignin-based industry. Metab Eng. 2022;71:13–41. 10.1016/j.ymben.2021.11.011 34864214

[5] Jin Y, Araki T, Kamimura N, Masai E, Nakamura M, Michinobu T. Biodegradable and wood adhesive polyesters based on lignin-derived 2-pyrone-4,6-dicarboxylic acid. RSC Sustain. 2024;2(7):1985–93.

[6] Bito M, Otsuka Y, Nakamura M, Masai E, Katayama Y, Shigehara K, et al. Unique complexation behavior of alkali metal ions and 2-pyrone-4,6-dicarboxylic acid (PDC) obtained from a metabolic intermediate of lignin. Waste Biomass Valoriz. 2019;10(5):1261–5.

[7] Michinobu T, Bito M, Yamada Y, Katayama Y, Noguchi K, Masai E, et al. Molecular properties of 2-pyrone-4,6-dicarboxylic acid (PDC) as a stable metabolic intermediate of lignin isolated by fractional precipitation with Na^+^ ion. Bull Chem Soc Jpn. 2007;80(12):2436–42.

[8] Otsuka Y, Araki T, Suzuki Y, Nakamura M, Kamimura N, Masai E. High-level production of 2-pyrone-4,6-dicarboxylic acid from vanillic acid as a lignin-related aromatic compound by metabolically engineered fermentation to realize industrial valorization processes of lignin. Bioresour Technol. 2023;377:128956. 10.1016/j.biortech.2023.128956 36965585

[9] Notonier S, Werner AZ, Kuatsjah E, Dumalo L, Abraham PE, Hatmaker EA, et al. Metabolism of syringyl lignin-derived compounds in *Pseudomonas putida* enables convergent production of 2-pyrone-4,6-dicarboxylic acid. Metab Eng. 2021;65:111–22. 10.1016/j.ymben.2021.02.005 33741529

[10] Lee S, Jeon BW, Seong JY, Lee I, Song HM, Ryu MH, et al. Efficient biological funneling of lignin into 2-pyrone-4,6-dicarboxylic acid via electrocatalytic depolymerization and genetically engineered *Pseudomonas putida* KT2440. Int J Biol Macromol. 2025;306:141657. 10.1016/j.ijbiomac.2025.141657 40032124

[11] Omolabake S, Hofsommer DT, Mains KM, Palumbo CT, Rigo D, Werner AZ, et al. Cu-catalyzed aerobic oxidative C─C cleavage in lignin-derived oligomers and biological funneling of the monomeric products. Angew Chem Int Ed Engl. 2026;65(2):e15588. 10.1002/anie.202515588 PMC12790356 41243566

[12] Kim B, Perez JM, Karlen SD, Coplien J, Donohue TJ, Noguera DR. Achieving high productivity of 2-pyrone-4,6-dicarboxylic acid from aqueous aromatic streams with *Novosphingobium aromaticivorans*. Green Chem. 2024;26(13):7997–8009.

[13] Perez JM, Sener C, Misra S, Umana GE, Coplien J, Haak D, et al. Integrating lignin depolymerization with microbial funneling processes using agronomically relevant feedstocks. Green Chem. 2022;24(7):2795–811.

[14] Kamimura N, Endo M, Nakamura B, Akiyama T, Kato R, Kawazoe M, et al. Platform potential of *Sphingobium lignivorans* SYK-6 for Lignin valorization via biological funneling. J Agric Food Chem. 2026;74(12):10406–15. 10.1021/acs.jafc.5c16625 PMC13047686 41873666

[15] Kamimura N, Takahashi K, Mori K, Araki T, Fujita M, Higuchi Y, et al. Bacterial catabolism of lignin-derived aromatics: new findings in a recent decade: update on bacterial lignin catabolism. Environ Microbiol Rep. 2017;9(6):679–705. 10.1111/1758-2229.12597 29052962

[16] Kawazoe M, Kato R, Fujita M, Hishiyama S, Kamimura N, Masai E. Precise transcription control drives optimized catabolism of *β*-5-type lignin-derived dimers in *Sphingobium lignivorans* SYK-6. J Agric Food Chem. 2025;73(37):23535–47. 10.1021/acs.jafc.5c06878 PMC12447483 40908941

[17] Mori K, Niinuma K, Fujita M, Kamimura N, Masai E. DdvK, a novel major facilitator superfamily transporter essential for 5,5′-dehydrodivanillate uptake by *Sphingobium* sp. strain SYK-6. Appl Environ Microbiol. 2018;84(20):e01314–01318. 10.1128/AEM.01314-18 PMC6182893 30120118

[18] Kato R, Kuatsjah E, Fujita M, Bleem AC, Hishiyama S, Katahira R, et al. Metabolic modification of *Sphingobium lignivorans* SYK-6 for lignin valorization through the discovery of an unusual transcriptional repressor of lignin-derived dimer catabolism. Green Chem. 2025;27(5):1540–55.

[19] De Paepe B, Maertens J, Vanholme B, De Mey M. Modularization and response curve engineering of a naringenin-responsive transcriptional biosensor. ACS Synth Biol. 2018;7(5):1303–14. 10.1021/acssynbio.7b00419 29688705

[20] Chiang AJ, Hasty J. Design of synthetic bacterial biosensors. Curr Opin Microbiol. 2023;76:102380. 10.1016/j.mib.2023.102380 37703812

[21] Wang K, Pan X, Yang T, Rao Z. Efficient production of salicylic acid through CmeR-P_cmeO_ biosensor-assisted multiplexing pathway optimization in *Escherichia coli*. Biotechnol Biofuels Bioprod. 2025;18(1):40. 10.1186/s13068-025-02637-2 PMC11954222 40156043

[22] Wu J, Jiang P, Chen W, Xiong D, Huang L, Jia J, et al. Design and application of a lactulose biosensor. Sci Rep. 2017;7(1):45994. 10.1038/srep45994 PMC5384092 28387245

[23] Zhang X, He Y, Wu Z, Liu G, Tao Y, Jin J-M, et al. Whole-cell biosensors aid exploration of vanillin transmembrane transport. J Agric Food Chem. 2021;69(10):3114–23. 10.1021/acs.jafc.0c07886 33666081

[24] Alvarez-Gonzalez G, Dixon N. Genetically encoded biosensors for lignocellulose valorization. Biotechnol Biofuels. 2019;12(1):246. 10.1186/s13068-019-1585-6 PMC6792243 31636705

[25] Zhao Z, Zhu R, Shi X, Yang F, Xu M, Shao M, et al. Combining biosensor and metabolic network optimization strategies for enhanced L-threonine production in *Escherichia coli*. Biotechnol Biof Bioprod. 2025;18(1):37. 10.1186/s13068-025-02640-7 PMC11938683 40134016

[26] Kamimura N, Aoyama T, Yoshida R, Takahashi K, Kasai D, Abe T, et al. Characterization of the protocatechuate 4,5-cleavage pathway operon in *Comamonas* sp. strain E6 and discovery of a novel pathway gene. Appl Environ Microbiol. 2010;76(24):8093–101. 10.1128/AEM.01863-10 PMC3008244 20952641

[27] Araki T, Umeda S, Kamimura N, Kasai D, Kumano S, Abe T, et al. Regulation of vanillate and syringate catabolism by a MarR-type transcriptional regulator DesR in *Sphingobium* sp. SYK-6. Sci Rep. 2019;9(1):18036. 10.1038/s41598-019-54490-7 PMC6888825 31792252

[28] Otsuka Y, Nakamura M, Shigehara K, Sugimura K, Masai E, Ohara S, et al. Efficient production of 2-pyrone 4,6-dicarboxylic acid as a novel polymer-based material from protocatechuate by microbial function. Appl Microbiol Biotechnol. 2006;71(5):608–14. 10.1007/s00253-005-0203-7 16322989

[29] Magara K, Kubo S. Factors affecting the yield of phenolic monomers in an oxygen-soda anthraquinone cooking. Jpn TAPPI J. 2019;73(3):249–58.

[30] Altschul SF, Gish W, Miller W, Myers EW, Lipman DJ. Basic local alignment search tool. J Mol Biol. 1990;215(3):403–10. 10.1016/S0022-2836(05)80360-2 2231712

[31] Li W, Cowley A, Uludag M, Gur T, McWilliam H, Squizzato S, et al. The EMBL-EBI bioinformatics web and programmatic tools framework. Nucleic Acids Res. 2015;43(W1):W580–4. 10.1093/nar/gkv279 PMC4489272 25845596

[32] Tamura K, Stecher G, Kumar S. MEGA11: molecular evolutionary genetics analysis version 11. Mol Biol Evol. 2021;38(7):3022–7. 10.1093/molbev/msab120 PMC8233496 33892491

[33] Zerbino DR, Birney E. Velvet: algorithms for de novo short read assembly using de Bruijn graphs. Genome Res. 2008;18(5):821–9. 10.1101/gr.074492.107 PMC2336801 18349386

[34] Tanizawa Y, Fujisawa T, Kaminuma E, Nakamura Y, Arita M. DFAST and DAGA: web-based integrated genome annotation tools and resources. Biosci Microbiota Food Health. 2016;35(4):173–84. 10.12938/bmfh.16-003 PMC5107635 27867804

[35] Hosoya T, Yamamoto K, Miyafuji H, Yamada T. Selective production of bio-based aromatics by aerobic oxidation of native soft wood lignin in tetrabutylammonium hydroxide. RSC Adv. 2020;10(33):19199–210. 10.1039/d0ra03420g PMC9054073 35515466

[36] Maeda M, Hosoya T, Yoshioka K, Miyafuji H, Ohno H, Yamada T. Vanillin production from native softwood lignin in the presence of tetrabutylammonium ion. J Wood Sci. 2018;64(6):810–5.

[37] Weeda EP, Holland CM, Behaghel de Bueren J, Yuan Z, Alherech M, Coplien J, et al. O_2_-permeable membrane reactor for continuous oxidative depolymerization of lignin. Joule. 2024;8(12):3336–46.

[38] Wang G, Øzmerih S, Guerreiro R, Meireles AC, Carolas A, Milne N, et al. Improvement of *cis,cis*-muconic acid production in *Saccharomyces cerevisiae* through biosensor-aided genome engineering. ACS Synth Biol. 2020;9(3):634–46. 10.1021/acssynbio.9b00477 PMC8457548 32058699

[39] Yu H, Wang N, Huo W, Zhang Y, Zhang W, Yang Y, et al. Establishment of BmoR-based biosensor to screen isobutanol overproducer. Microb Cell Fact. 2019;18(1):30. 10.1186/s12934-019-1084-2 PMC6366067 30732651

[40] Han G, Xu N, Sun X, Chen J, Chen C, Wang Q. Improvement of L-valine production by atmospheric and room temperature plasma mutagenesis and high-throughput screening in *Corynebacterium glutamicum*. ACS Omega. 2020;5(10):4751–8. 10.1021/acsomega.9b02747 PMC7081258 32201760

[41] Zhang X, Zhang X, Xu G, Zhang X, Shi J, Xu Z. Integration of ARTP mutagenesis with biosensor-mediated high-throughput screening to improve L-serine yield in *Corynebacterium glutamicum*. Appl Microbiol Biotechnol. 2018;102(14):5939–51. 10.1007/s00253-018-9025-2 29725721

[42] Wang M, Wang H, Gao C, Wei W, Liu J, Chen X, et al. Efficient production of protocatechuic acid using systems engineering of *Escherichia coli*. Metab Eng. 2024;82:134–46. 10.1016/j.ymben.2024.02.003 38369051

[43] Pardo I, Jha RK, Bermel RE, Bratti F, Gaddis M, McIntyre E, et al. Gene amplification, laboratory evolution, and biosensor screening reveal MucK as a terephthalic acid transporter in *Acinetobacter baylyi* ADP1. Metab Eng. 2020;62:260–74. 10.1016/j.ymben.2020.09.009 32979486

[44] Ho JCH, Pawar SV, Hallam SJ, Yadav VG. An improved whole-cell biosensor for the discovery of lignin-transforming enzymes in functional metagenomic screens. ACS Synth Biol. 2018;7(2):392–8. 10.1021/acssynbio.7b00412 29182267

[45] Uchiyama T, Miyazaki K. Product-induced gene expression, a product-responsive reporter assay used to screen metagenomic libraries for enzyme-encoding genes. Appl Environ Microbiol. 2010;76(21):7029–35. 10.1128/AEM.00464-10 PMC2976229 20833789

[46] Han Y, Simmons BA, Singh S. Perspective on oligomeric products from lignin depolymerization: their generation, identification, and further valorization. Ind Chem Mater. 2023;1(2):207–23.

[47] Dou Y, Zhang J, Gong X, Gan Q, Yu S, Yan Y. Detection of environmental pollutants using transcription factor-based whole-cell biosensors. Biotechnol Environ. 2025;2(1):16. 10.1186/s44314-025-00032-7 PMC12665635 41333561

[48] Tanniche I, Behkam B. Engineered live bacteria as disease detection and diagnosis tools. J Biol Eng. 2023;17(1):65. 10.1186/s13036-023-00379-z PMC10598922 37875910

[49] Li M, Chen Z, Huo Y-X. Application evaluation and performance-directed improvement of the native and engineered biosensors. ACS Sens. 2024;9(10):5002–24. 10.1021/acssensors.4c01072 39392681

[50] Leonard AC, Whitehead TA. Design and engineering of genetically encoded protein biosensors for small molecules. Curr Opin Biotechnol. 2022;78:102787. 10.1016/j.copbio.2022.102787 PMC12543005 36058141

[51] Nishikawa KK, Chen J, Acheson JF, Harbaugh SV, Huss P, Frenkel M, et al. Highly multiplexed design of an allosteric transcription factor to sense new ligands. Nat Commun. 2024;15(1):10001. 10.1038/s41467-024-54260-8 PMC11577015 39562775

[52] Sana B, Chia KHB, Raghavan SS, Ramalingam B, Nagarajan N, Seayad J, et al. Development of a genetically programed vanillin-sensing bacterium for high-throughput screening of lignin-degrading enzyme libraries. Biotechnol Biofuels. 2017;10(1):32. 10.1186/s13068-017-0720-5 PMC5291986 28174601

[53] Kunjapur AM, Prather KLJ. Development of a vanillate biosensor for the vanillin biosynthesis pathway in *E. coli*. ACS Synth Biol. 2019;8(9):1958–67. 10.1021/acssynbio.9b00071 31461264

[54] Bentley GJ, Narayanan N, Jha RK, Salvachúa D, Elmore JR, Peabody GL, et al. Engineering glucose metabolism for enhanced muconic acid production in *Pseudomonas putida* KT2440. Metab Eng. 2020;59:64–75. 10.1016/j.ymben.2020.01.001 31931111

[55] Baugh AC, Momany C, Neidle EL. Versatility and complexity: common and uncommon facets of LysR-type transcriptional regulators. Annu Rev Microbiol. 2023;77:317–39. 10.1146/annurev-micro-050323-040543 37285554

[56] Demeester W, De Paepe B, De Mey M. Fundamentals and exceptions of the LysR-type transcriptional regulators. ACS Synth Biol. 2024;13(10):3069–92. 10.1021/acssynbio.4c00219 PMC11495319 39306765

[57] Snoek T, Chaberski EK, Ambri F, Kol S, Bjørn SP, Pang B, et al. Evolution-guided engineering of small-molecule biosensors. Nucl Acids Res. 2020;48(1):e3. 10.1093/nar/gkz954 PMC6943132 31777933

[58] Li Q, Ren H, Liao Z, Xia S, Sun X. High throughput screening of transcription factor LysG for constructing a better lysine biosensor. Biosensors (Basel). 2024;14(10):455. 10.3390/bios14100455 PMC11506072 39451669

