# Supplementary material for: A 2-pyrone-4,6-dicarboxylic acid biosensor for lignin valorization based on a newly identified transcriptional regulatory system
Source: Biotechnol Biofuels Bioprod. 2026 Jul 23;19:83. doi: 10.1186/s13068-026-02797-9 (PMC13681972; doi:10.1186/s13068-026-02797-9)
Supplement: Supplementary file 1 [file 13068_2026_2797_MOESM1_ESM.pdf]

Additional File 1

A 2-pyrone-4,6-dicarboxylic acid biosensor for lignin valorization based on a newly identified transcriptional regulatory system

Kenta Tanatani[a†], Hyo Suzuki[a†], Masaya Fujita[a], Takuma Araki[b], Yuzo Suzuki[b], Tsuyoshi Michinobu[c], Yuichiro Otsuka[b], Masaya Nakamura[b], Eiji Masai[a], Naofumi Kamimura[a*]

[a]Department of Materials Science and Bioengineering, Nagaoka University of Technology, Nagaoka, Niigata, Japan.
[b]Department of Forest Resource Chemistry, Forestry and Forest Products Research Institute, Tsukuba, Ibaraki, Japan
[c]Department of Materials Science and Engineering, Institute of Science Tokyo, Meguro-ku, Tokyo, Japan.

[†]Kenta Tanatani and Hyo Suzuki contributed equally to this work.
*Correspondence:
Naofumi Kamimura
Department of Materials Science and Bioengineering, Nagaoka University of Technology, Nagaoka, Niigata 940-2188, Japan
E-mail: zkami@vos.nagaokaut.ac.jp
TEL: +81 258 47 9402

Table S1. Strains and plasmids used in this study

Strains and Plasmids	Relevant characteristic(s)[a,b]	References or Source
Strains		
Sphingobium lignivorans SYK-6	Wild type; Nal^r Sm^r	[1]
SME002-3 (Δ*ligI*)	SYK-6 derivative; ΔSLG_12570 (*ligI*)	[2]
SME257 (Δ*ligAB*)	SYK-6 derivative; ΔSLG_12500 (*ligA*), SLG_12510 (*ligB*)	[3]
Cupriavidus sp. 8B	Wild type; PDC+	This study
Burkholderia sp. 3C	Wild type; PDC+	This study
Paraburkholderia sp. 2C	Wild type; PDC+	This study
Pseudomonas sp. 3A	Wild type; PDC+	This study
Novosphingobium sp. 11B	Wild type; PDC+	This study
Pseudarthrobacter sp. 8C	Wild type; PDC+	This study
Comamonas sp. E6	Wild type; Amp^r	[4]
Escherichia coli		
NEB 10-beta	Δ(*ara-leu*) *7697 araD139 fhuA* Δ*lacX74 galK16 galE15 e14-* *ϕ80dlacZ* Δ*M15 racA1 relA1 endA1 nupG rpsL* (Sm^r) *rph spoT1* Δ(*mrr-hsdRMS-mcrBC*)	New England Biolabs
HB101	*recA13 supE44 hsd20 ara-14 proA2 lacY1 galK2 rpsL20 xyl-5 mtl-1*	[5]
Plasmids		
pRK2013	Tra^+ Mob^+ ColE1 replicon; Km^r	[6]
pSEVA231	pBBR1 broad-host-range vector; Km^r	[7]
pPNS	pSEVA231 with a codon optimized *mNeonGreen.*	This study
pCup1	pPNS with P_{CS8_000090} and codon optimized CS8_000100	This study
pCup2	pPNS with P_{CS8_000090} and codon optimized CS8_000110	This study
pCup3	pPNS with P_{CS8_000120} and codon optimized CS8_000100	This study
pCup4	pPNS with P_{CS8_000120} and codon optimized CS8_000110	This study
pCup0	pPNS with P_{CS8_000090}	This study
pBur	pPNS with P_{BGC_43260} and codon optimized BGC_43250	This study
pPar	pPNS with P_{PBS_63070} and codon optimized PBS_63060	This study
pPse	pPNS with P_{PS3A_06990} and codon optimized PS3A_06980	This study
pNov	pPNS with P_{NRB_04780} and codon optimized NRB_04800	This study

[a]Abbreviations: Nal^r, Sm^r, Amp^r, and Km^r, resistance to nalidixic acid, streptomycin, ampicillin, and kanamycin, respectively.

[b]PDC+, able to assimilate PDC as a sole carbon source.

Table S2. Nucleotide sequences of primers used in this study

Primers	Nucleotide sequence (5' to 3')
For construction of plasmids	
pPNS	
mNeonGreen_F	GTCGACTCTAGAGGATCCCCGATATCAAAGGAGGTATATATAATGGTGTCCAAAGGCGAAGA
mNeonGreen_R	CGAATTCGAGCTCGGTACCCTCACTTATAGAGCTCGTCCA
pCup1, pCup2	
CS8_000090promoter_F	GAGCGGATACTTATGTCAAACGTCCAGGTCCTGCGACGCG
CS8_000090promoter_R	TTATATATACCTCCTTTGATGATGTGGCTGGGCTCTATTG
pCup3, pCup4	
CS8_000120promoter_F	GAGCGGATACTTATGTCAAACGCCGAGGGCGATGTTGCTG
CS8_000120promoter_R	TTATATATACCTCCTTTGATGCGACTGATTATCGAGACTC
pCup0	
only CS8_000090promoter	GACTCTAGAGGATCCCCGATCCGCTGACCGTCCCCTTCAA
CS8_000090promoter_R	TTATATATACCTCCTTTGATGATGTGGCTGGGCTCTATTG
pBur	
BGC_43260promoter_F	GAGCGGATACTTATGTCAAATGATGCCGATGCCGAAATGG
BGC_43260promoter_R	TTATATATACCTCCTTTGATCGGCTGTCTCGTGGCTGGCC
pPar	
PBS_63070promoter_F	GAGCGGATACTTATGTCAAACGTCCAGGTCCTGCGACGCG
PBS_63070promoter_R	TTATATATACCTCCTTTGATCGAGGTATGACTTGTCTCAT
pPse	
PS3A_06990promoter_F	GAGCGGATACTTATGTCAAAGCGATGGCTATCGGGTCTAT
PS3A_06990promoter_R	TTATATATACCTCCTTTGATCTCGAATCCTTTTTTGTTGT
pNov	
NRB_04780promoter_F	GAGCGGATACTTATGTCAAAGTCCGCCGAATGTCTGTTGT
NRB_04780promoter_R	TTATATATACCTCCTTTGATGATGCGTGTTTTCCGTCGGA
For 16S rRNA gene sequence	
533f	GTGCCAGCAGCCGCGGTAA
1525r	AAAGGAGGTGATCCAGCC
For verifying PCR amplification	
F24-2	TCATTACTGGATCTATCAACAGG
R24	AGCGGATAACAATTTCACACAGGA
mNeonGreen seq	CAGCTCATGGGTCGCTGGAA

Table S3. PDC hydrolase genes identified in each strain

Strains	Locus tag	Most similar PDC hydrolase[a]	Identity[b]
Cupriavidus sp. 8B	CS8_000160	ProL	71.0%
	CS8_000090	ProL	45.2%
	CS8_042680	Saro_2819	35.6%
Burkholderia sp. 3C	BGC_43260	ProL	46.1%
	BGC_19840	PcmC	39.6%
	BGC_43200	PmdD	32.0%
	BGC_13820	LigI	28.8%
	BGC_37800	Saro_2819	31.7%
Paraburkholderia sp. 2C	PBS_63070	ProL	44.6%
	PBS_10520	ProL	30.5%
	PBS_06260	PmdD	32.0%
Pseudomonas sp. 3A	PS3A_06990	PcmC	45.5%
	PS3A_18270	PmdD	35.2%
	PS3A_38600	ProL	36.8%
	PS3A_38580	PmdD	20.1%
	PS3A_07090	Saro_2819	30.1%
	PS3A_52280	PcmC	20.4%
Novosphingobium sp. 11B	NRB_04770	PmdD	67.2%

[a]Known PDC hydrolases: ProL from *Pseudomonas straminea* (accession number, BAD04056.1); PmdD from *Comamonas* sp. E6 (GAO68735.1); PcmC from *Arthrobacter keyseri* (AAK16526); Saro_2819 from *Novosphingobium aromaticivorans* DSM 12444 (ABD27255); LigI from *Sphingobium lignivorans* SYK-6 (BAK65932.1).

[b]Amino acid sequence identity with known PDC hydrolases, calculated using the EMBOSS-Needle program (https://www.ebi.ac.uk/jdispatcher/psa/emboss_needle).

Table S4. Construction of reporter plasmids and sequences of synthetic genes

Regulatory gene or promoter	Synthesized nucleotide sequence (5' to 3')[a]
pCup1<br>Regulatory gene<br>*Cupriavidus* sp. 8B<br>CS8_000100	GACTCTAGAGGATCCCCGATTCACATCTCATTTGGCTGCATTTCGCCTGGATTACGGTCGGTCGTATACAGGGGCAGCA<br>CGGCCGTCTCGATAAGGCCGATGGCCTCCATGGCCAGATGCGACAGCGTGTGCTGGGAGCTGGTCGCTATCGCGAGG<br>CGAGTATGCAGCGTTGGCTGGGCGATCCGCGTGACCGAGAACGGCCGCTTCAGCGGATCCGTGGCCGGCGCGTTGAT<br>GGGCAGGACCGCGTAGCCGTAACCCTCGCAGACCAGGTCAAGGATGGCGCCGACCGCATCGACCTCGAGGGCGATC<br>GCGAGCTTGACTCCTTCCTCAGCCGCCCGCGCCTCAACGATCTGGCGCAGGGCATGCATCCGGCCCGGGATCACCAG<br>CGGATACCGGCCGATATCCTTGAGCCGGATCTGCTCTGGGGCCTGGCCCCCGGTGGAGCCTCCCATGCCGATGAGGAA<br>CAGTTCCTCTTCGAAGACAGCCCGTTTCTCGACGAGCGCCGTGAGCGACGTATCATAGAGGACGGCAGCATCGATCC<br>GGCCCATCATCAGCCACTCCATGAGGTACGTCGAGAGGCCCTCCGCAACGCTGATGGTGGCATCCGGGAACCGCTGG<br>CGGAACTGGCGGATGAGCTCCTGAGTCGCAACCTTGGCAAAGCTGGGGGCCAGGCCAATCGAGAAGTGGCCGCTCA<br>CGGTCCCTTTCAGCTGTCGAAGATCGTGACGCGCGCGTTCCGCCTGCAGAAGCATGCCTTTCGCATGCCCCAGCATGA<br>CCTGACCGGCATCCGTCGCAAGGACCCCACGGCCATTACGGACCAGAAGGGTCTGGCGCAGTTCGACTTCCAGCTGA<br>CGGACCAGGCGGCTCAGGGTGGGCTGGTTCGTGTCCAGTATCGTCGCGGCACGGGTGAAGGAGCCCAGTTCCACGA<br>CATGGACAAAGGCCTCAAGCTGCTTAAGTTCCATTATATATACCTCCTTTATTGTATCATGAGCGGATACTTATGTCAAA
Promoter<br>*Cupriavidus* sp. 8B<br>P_{CS8_000090}	CCGCTGACCGTCCCCTTCAATTGCCGAAGATCGTGCCGGGCACGCTCGGCCTGCAGCAGCATCCCCTTCGCATGGCCC<br>AGCATGACCTGCCCCGCATCGGTTGCCAGGACGCCGCGCCCGTTCCGCACGAGCAGGGTCTGCCTAAGTTCCACCTC<br>GAGCTGTCGCACGAGGCGGCTCAAGGTCGGCTGATTGGTATCGAGGATCGTGGCGGCGCGCGTGAAGCTCCCCAGCT<br>CCACTACGTGCACAAAGGCCTCGAGCTGCTTCAGTTCCATTTCGGATCCTATACAAATTTCGTATATCAGCTAGTCGGA<br>TATGTCGATTGTATCGGTGAACTCGAATCGCCACAATGATCCTCAATCGCTGTCAGGCCACGCCATTCTCAATAGAGCC<br>CAGCCACATC-(ATG, CS8_000090)
pCup2<br>Regulatory gene<br>*Cupriavidus* sp. 8B<br>CS8_000110	GACTCTAGAGGATCCCCGATTCAGGCCGCTTCCCGGCCGTGGAGGACAGCCCGGGTCGTCTGCAGGATGACGTCGAGCATGC<br>TCTGGTGCGACCGCGTGCTCGGGCGATGGCTCGACACGGCGAGGACCAGTCGCATTCGGAGGCCGGGGGAGCTGATGGGTCG<br>CGCCACCAGACGGTCGGCGTGAGTCGATTTCAGGAGCGCGTTTATCGGGAGGACGGCCCCTCCGGCGCCATCCGCCACGAGA<br>TCCAGGAGCGAGGACACGCTATCGATTTCGAGCGCAATCGTGGGCACAAGGCCGACGTCGGTAAGCCGCGTCTCCACCTTCA<br>TACGCATGGCATTCGGGCGGCTGGGTATCACCAGCGGAGTCTGCGCGAGTTCCTCGAGAGTGACCGGCGCCGTGGCATTCGA<br>GGACCCGCCAAGCTCTATGAGGAAAAGATCTTCCTCACAGACTTCCGTGAATTCGACGCCCGACGCCATCGCGTTCCCGGGG<br>AATTCATACAGGAGGGCCGCATCGAGGCGGCCGTTAAGGAGGGACTCCTGCATGGCGGTCGACAGGCCTTCGCGGACGCTCA<br>GGACCGTGCCTGGCAGCGCCGTCCGGAACTGGCGGATGAGCGGAACGATAAGTATTCGCGAGAGCGAGGGTGGGACGCCGA<br>GCGCTATATTGGAGCCCGCGGCGCCGCGGAGGCGCGCCAGTTCCTCTTTAGTATGCGCGAACTGTCGAAGCAGTCCCCGGCC<br>ATGCTCAAGGAGCAGTTTGCCCGCCTCGGTCGTGGTCACACCGCGGCCGTTACGGATGAGCAGATTTTGGCGCAGCTCCACCT<br>CGAGAAGCCGAACCTGTCGCGAGAGCGCCGGCTGGGCGACATTGAGGACCACGGACGCTCGGGTGAAGCTCGAAAGTTCCG<br>CGACATGCACGAAATACTCGAGCTGCTTTATATCCATTATATATACCTCCTTTATTGTATCATGAGCGGATACTTATGTCAAA
Promoter<br>*Cupriavidus* sp. 8B<br>P_{CS8_000090}	CCGCTGACCGTCCCCTTCAATTGCCGAAGATCGTGCCGGGCACGCTCGGCCTGCAGCAGCATCCCCTTCGCATGGCCC<br>AGCATGACCTGCCCCGCATCGGTTGCCAGGACGCCGCGCCCGTTCCGCACGAGCAGGGTCTGCCTAAGTTCCACCTC<br>GAGCTGTCGCACGAGGCGGCTCAAGGTCGGCTGATTGGTATCGAGGATCGTGGCGGCGCGCGTGAAGCTCCCCAGCT<br>CCACTACGTGCACAAAGGCCTCGAGCTGCTTCAGTTCCATTTCGGATCCTATACAAATTTCGTATATCAGCTAGTCGGA<br>TATGTCGATTGTATCGGTGAACTCGAATCGCCACAATGATCCTCAATCGCTGTCAGGCCACGCCATTCTCAATAGAGCC<br>CAGCCACATC-(ATG, CS8_000090)
pCup3<br>Regulatory gene<br>*Cupriavidus* sp. 8B<br>CS8_000100	GACTCTAGAGGATCCCCGATTCACATCTCATTTGGCTGCATTTCGCCTGGATTACGGTCGGTCGTATACAGGGGCAGCACGGC<br>CGTCTCGATAAGGCCGATGGCCTCCATGGCCAGATGCGACAGCGTGTGCTGGGAGCTGGTCGCTATCGCGAGGCGAGTATGC<br>AGCGTTGGCTGGGCGATCCGCGTGACCGAGAACGGCCGCTTCAGCGGATCCGTGGCCGGCGCGTTGATGGGCAGGACCGCGT<br>AGCCGTAACCCTCGCAGACCAGGTCAAGGATGGCGCCGACCGCATCGACCTCGAGGGCGATCGCGAGCTTGACTCCTTCCTC<br>AGCCGCCCGCGCCTCAACGATCTGGCGCAGGGCATGCATCCGGCCCGGGATCACCAGCGGATACCGGCCGATATCCTTGAGC<br>CGGATCTGCTCTGGGGCCTGGCCCCCGGTGGAGCCTCCCATGCCGATGAGGAACAGTTCCTCTTCGAAGACAGCCCGTTTCTC<br>GACGAGCGCCGTGAGCGACGTATCATAGAGGACGGCAGCATCGATCCGGCCCATCATCAGCCACTCCATGAGGTACGTCGAG<br>AGGCCCTCCGCAACGCTGATGGTGGCATCCGGGAACCGCTGGCGGAACTGGCGGATGAGCTCCTGAGTCGCAACCTTGGCAA<br>AGCTGGGGGCCAGGCCAATCGAGAAGTGGCCGCTCACGGTCCCTTTCAGCTGTCGAAGATCGTGACGCGCGCGTTCCGCCTG<br>CAGAAGCATGCCTTTCGCATGCCCCAGCATGACCTGACCGGCATCCGTCGCAAGGACCCCACGGCCATTACGGACCAGAAGG<br>GTCTGGCGCAGTTCGACTTCCAGCTGACGGACCAGGCGGCTCAGGGTGGGCTGGTTCGTGTCCAGTATCGTCGCGGCACGGGT<br>GAAGGAGCCCAGTTCCACGACATGGACAAAGGCCTCAAGCTGCTTAAGTTCCATTATATATACCTCCTTTATTGTATCATGAG<br>CGGATACTTATGTCAAA
Promoter<br>*Cupriavidus* sp. 8B<br>P_{CS8_000120}	CGCCGAGGGCGATGTTGCTGCCTGCGGCACCGCGCAGCCGCGCTAATTCTTCCTTGGTATGGGCGAATTGGCGCAGTA<br>GCCCGCGGCCATGCTCCAGTAGCAGCTTCCCGGCCTCGGTGGTGGTGACCCCGCGTCCGTTGCGGATCAACAGGTTC<br>TGCCGCAACTCGACCTCCAGCAGCCTGACTTGCCGACTAAGGGCAGGTTGAGCCACATTTAGCACGACGGAAGCACG<br>GGTAAAGCTGCTCAATTCGGCAACGTGCACGAAGTATTCGAGTTGTTTGATGTCCATGGCATTGGGTTGCAGCGCAGC<br>AAATCACGCCTTGCGTTATAGCAGAATGATATATCTGCTAGTGGGATTGTGCGCTGTTTCAATCTTGGCCGAGTCTCGAT<br>AATCAGTCGC-(ATG, CS8_000120)

Table S4. –*Continued*

**pCup4**	
Regulatory gene<br>*Cupriavidus* sp. 8B<br>CS8_000110	GACTCTAGAGGATCCCCGATTCAGGCCGCTTCCCGGCCGTGGAGGACAGCCCGGGTCGTCTGCAGGATGACGTCGAGCATGC<br>TCTGGTGCGACCGCGTGCTCGGGCGATGGCTCGACACGGCGAGGACCAGTCGCATTCGGAGGCCGGGGGAGCTGATGGGTCG<br>CGCCACCAGACGGTCGGCGTGAGTCGATTTCAGGAGCGCGTTTATCGGGAGGACGGCCCCTCCGGCGCCATCCGCCACGAGA<br>TCCAGGAGCGAGGACACGCTATCGATTTCGAGCGCAATCGTGGGCACAAGGCCGACGTCGGTAAGCCGCGTCTCCACCTTCA<br>TACGCATGGCATTCGGGCGGCTGGGTATCACCAGCGGAGTCTGCGCGAGTTCCTCGAGAGTGACCGGCGCCGTGGCATTCGA<br>GGACCCGCCAAGCTCTATGAGGAAAAGATCTTCCTCACAGACTTCCGTGAATTCGACGCCCGACGCCATCGCGTTCCCGGGG<br>AATTCATACAGGAGGGCCGCATCGAGGCGGCCGTTAAGGAGGGACTCCTGCATGGCGGTCGACAGGCCTTCGCGGACGCTCA<br>GGACCGTGCCTGGCAGCGCCGTCCGGAACTGGCGGATGAGCGGAACGATAAGTATTCGCGAGAGCGAGGGTGGGACGCCGA<br>GCGCTATATTGGAGCCCGCGGCGCCGCGGAGGCGCGCCAGTTCCTCTTTAGTATGCGCGAACTGTCGAAGCAGTCCCCGGCC<br>ATGCTCAAGGAGCAGTTTGCCCGCCTCGGTCGTGGTCACACCGCGGCCGTTACGGATGAGCAGATTTTGGCGCAGCTCCACCT<br>CGAGAAGCCGAACCTGTCGCGAGAGCGCCGGCTGGGCGACATTGAGGACCACGGACGCTCGGGTGAAGCTCGAAAGTTCCG<br>CGACATGCACGAAATACTCGAGCTGCTTTATATCCATTATATATACCTCCTTTATTGTATCATGAGCGGATACTTATGTCAAA
Promoter<br>*Cupriavidus* sp. 8B<br>P_{CS8_000120}	CGCCGAGGGCGATGTTGCTGCCTGCGGCACCGCGCAGCCGCGCTAATTCTTCCTTGGTATGGGCGAATTGGCGCAGTA<br>GCCCGCGGCCATGCTCCAGTAGCAGCTTCCCGGCCTCGGTGGTGGTGACCCCGCGTCCGTTGCGGATCAACAGGTTC<br>TGCCGCAACTCGACCTCCAGCAGCCTGACTTGCCGACTAAGGGCAGGTTGAGCCACATTTAGCACGACGGAAGCACG<br>GGTAAAGCTGCTCAATTCGGCAACGTGCACGAAGTATTCGAGTTGTTTGATGTCCATGGCATTGGGTTGCAGCGCAGC<br>AAATCACGCCTTGCGTTATAGCAGAATGATATATCTGCTAGTGGGATTGTGCGCTGTTTCAATCTTGGCCGAGTCTCGAT<br>AATCAGTCGC-(ATG, CS8_000120)
**pCup0**	
Promoter<br>*Cupriavidus* sp. 8B<br>P_{CS8_000090}	CCGCTGACCGTCCCCTTCAATTGCCGAAGATCGTGCCGGGCACGCTCGGCCTGCAGCAGCATCCCCTTCGCATGGCCC<br>AGCATGACCTGCCCCGCATCGGTTGCCAGGACGCCGCGCCCGTTCCGCACGAGCAGGGTCTGCCTAAGTTCCACCTC<br>GAGCTGTCGCACGAGGCGGCTCAAGGTCGGCTGATTGGTATCGAGGATCGTGGCGGCGCGCGTGAAGCTCCCCAGCT<br>CCACTACGTGCACAAAGGCCTCGAGCTGCTTCAGTTCCATTTCGGATCCTATACAAATTTCGTATATCAGCTAGTCGGA<br>TATGTCGATTGTATCGGTGAACTCGAATCGCCACAATGATCCTCAATCGCTGTCAGGCCACGCCATTCTCAATAGAGCC<br>CAGCCACATC-(ATG, CS8_000090)
**pBur**	
Regulatory gene<br>*Burkholderia* sp. 3C<br>BGC_43250	GACTCTAGAGGATCCCCGATTCAGCGCCCTCGGGTAGCGCCGTAAAGCGGGAGCACCTGGGCCTCAAGCATGGCGATGGCCT<br>GCCGGGCGAGCTGCGGCAGCGGGTGCCGGCGCGACGTCGCGATGGCGAGGCGGCTGTAAAGCGTCGGTTCCGTGATGCGCGT<br>GAGGCGGAAATGACCGGGCGGCAGATCCGCGGGGATGGCATTCAGGGAGAGCACGGCATGGCCGATACCTTCGCTGACCAG<br>GTCGAGTATGCTAGCGACACCGTCGACTTCGAGCTCTATGTTGAGACGTATCCCCTGTTCCGCGGCCGCGGCCTCCACCATGT<br>GGCGAATCGCGTGCATGCGCCCGGGGATGACGAGCGGATACCGGACTATCTCGCGGAGGGTAATGCTCTCGACCGGGGCCGC<br>ATGGCCTGGACCCACAAGAAACAGCTCTTCGGTGTGGATGGTGCGCTTGTCTATGGAGGGCGTGTCGAAAGTATCGTACAGG<br>ACCGCCACGTCTATACGGCCCATCATAAGCCACTCCGTCAGGTAGGTGCTCAGACCCTGAGCCACGGAGAGGGTGGCACCGG<br>GGAAGGTGCCTCGGAAATGGCGAACAAGCTCCAGGGTGCCGACGCGGGCGAACGAGGGCGTTATCCCTATGCCGAAGTGGC<br>CACCCAGCGTGCCTCGGAGCTCGTCGAGATCCTGGCTGGCGCGCTGCACCTGTTGCAGGAGACCTTTCGCATGCGCGAGCATG<br>CGCTGGCCGGCTGGCGTGAGAGTCACGCCACGGCCATTACGTTCGAGCAGGGTGTGGCGGAGCTCGACTTCAAGCTGCCGCA<br>CGAGGCGGGAGAGGGCGGGTTGGTTCGTATTCAGCGTGTTCGCGGCACGGGTGAAGGAGCCGAGTTCCGCGACATGGACGA<br>ACGCGTCGAGCTGTCGGAGGTCCATTATATATACCTCCTTTATTGTATCATGAGCGGATACTTATGTCAAA
Promoter<br>*Burkholderia* sp. 3C<br>P_{BGC_43260}	TGATGCCGATGCCGAAATGGCCGCCGAGCGTGCCGCGCAGTTCGTCGAGATCCTGCGAGGCGCGCTGCACCTGCTGC<br>AACAGCCCCTTGGCGTGCGCCAGCATGCGCTGGCCGGCCGGCGTCAGCGTCACGCCGCGCCCGTTGCGCTCCAGCAG<br>CGTGTGGCGCAGTTCCACCTCCAGTTGCCGCACCAGGCGGCTCAGGGCGGGTTGGTTGGTGTTCAGCGTGTTGGCGG<br>CGCGCGTGAAGCTGCCGAGTTCCGCCACATGGACGAATGCGTCGAGTTGCCGGAGATCCATGATCGAGATATGTGGA<br>ATGTGTATAACAGCTAGTTTGGTATGGCGATTGTAGCGAGGCAGCGGAGCCACCACAATGCACACCATTCCCGAACGA<br>CGGCCGCCGCGAGCGGCCAGCCACGAGACAGCCG-(ATG, BGC_43260)
**pPar**	
Regulatory gene<br>*Paraburkholderia* sp. 2C<br>PBS_63060	GACTCTAGAGGATCCCCGATTCAGTTAGCCGAGCCATAAAGCGGAAGGACCGAGGATTCCAGCATCGCGATGCTCTGTATAG<br>CCAGCTGGCTGAGCGTATGCTTGGTGGACGTCGCAATGGCGAGGCGGCTGAACAGCGTTGGCTCGCTTATGCGGAGGATGCG<br>GAAGTGCGGATCCTGAGCGCCTCCGCTGATGGCCTTCAGCGGAAGCACGGCATAGCCGATGCCTTCCTTCACCAGATCCAGA<br>ATGGACGCCACGCCATCCACTTCCAGCGCGATACGCAGTCGAACGCCCTGTTCGGCCGCGGCGGACTCCACCATGCGACGGA<br>TCGCGTGCATGCGGCCGGGGATTATGAGCGGATACTTGACGATGTCCCGCAGGGGCACCATGCTCGGGCCGGCTATGCACGG<br>CTGCGCCAGGGAGTCATCCGTGTCGGGGCCGATCAGGAACAGCTCTTCGGTGTGGACCGTGCGCTTATCGATCAGGGGCGTA<br>TCGAAGGTGTCATAGAGGACCGCCACATCGATTCGCCCCATCATCAGCCACTCGATAAGATGGGTGGAGAGGCCCTGCGCGA<br>CGCTAATCGTCGCGCCGGGAAAGGACTCCCGGAAGCTATGGACGAGTCGGTTGGTCGCAACTTTCGCGAAGGAGGGCGTGAC<br>GCCCACGCCGAAGTGGCCACCCGACGTGCCGCGAAGCTCGTCGAGGTCCTGGGACGCCCGCTGAACCTGTTGAAGGATTCCT<br>TTCGCGTGCGAGAGCATTCGCTGACCGGCCGAGGTCAGGCTGACACCACGGCCGTTCCGCTCGAGCAGATTGTGGCGGAGTT<br>CCACTTCCAGCTGGCGGACCAGTCGGCTCAGCGCGGGCTGGTTGGTGTCCAGCGTGATGGCCGCTCGGGTGAAGCTGCCGAG<br>CTCCGCGACGTGGACGAAAGCTTCGAGCTGCTTCAGATCCATTATATATACCTCCTTTATTGTATCATGAGCGGATACTTATGT<br>CAAA
Promoter<br>*Paraburkholderia* sp. 2C<br>P_{PBS_63070}	CGTCCAGGTCCTGCGACGCGCGCTGCACCTGCTGCAGAATCCCCTTCGCGTGCGAAAGCATCCGCTGGCCGGCCGAA<br>GTGAGCGATACGCCGCGGCCATTGCGCTCCAGCAGGTTATGACGCAATTCCACTTCGAGTTGCCGCACGAGGCGGCT<br>CAACGCCGGCTGGTTGGTGTCGAGCGTGATGGCCGCTCGTGTGAAGCTGCCTAGCTCCGCGACGTGCACGAAAGCTT<br>CGAGCTGTTTGAGGTCCATGCGGTCGAATCTATGTGAAATTCACATATCAGCTAGTTGGATATGACGATTGTAGGCGAA<br>CGGCCACGCTCTCACAATTCATTCGCACGCTAGAAAGACGTGTCGCGCACCCGCGCAGATGTCGCCGAACATGAGAC<br>AAGTCATACCTCG-(ATG, PBS_63070)

Table S4. –*Continued*

pPse	
Regulatory gene<br>*Pseudomonas* sp. 3A<br>PS3A_06980	GACTCTAGAGGATCCCCGATTCACGGCTGGCTCAGGAGTTGTTCGTTGATGACCGTCTGGTCACGTCCCCGCGCGACAAGCCA<br>GCGGTGGAAATCGCGGCAATGCGCCTTGTCGAAGGCGTCCCGCGCGCCGACGAGGAAATAGGACGACGGCATTTTCAGTCCC<br>TGCGCGAATGGAATGACCAGCCGACCGTCCGCGACAGCATCCGTGATCATGGAGCACTGCGCCAGGACCACCCCCTGTCCGG<br>CGATCGCAGCTTGTATGGCCATGGAGCTCAGGCTATACACTCGATAGCCATCGCGCAGATGGGCGCAGTCCACCTGTTGGGC<br>AGCGAACCAATCGCGCCAGGACGGGGGCGAGGCAAATTTCGGGAGCCAATCGACGCTAAGCAGGGGGTAGGCCTGGAGATC<br>AGCCGGAGTCCGGAGGGGGAGATCGGCCCGCAGAAGGTGCGGCGAACAGACCGGGACCACATCGTCCCGGAACAGTTCAAC<br>CGCGTGTTCGGTCGCGGCGTGATCGCCATAGGTGACGCGGAAGTCAATTTCGTACCCATCCGCGGCTGGTTCGGCGTGGCTGC<br>CGTCAAGGAACAGGTTAAGCTCCGGGTGTTCGGACTGCCACGCATAAATCAGTGGCGCCAGCCAATCGGACAGAAGGGATGG<br>GAGGGCGGAGATGCGGAGATTGGACGCGCTTCGCGACCGCTCGATTTCCACCTGGGCAATGCGCAGCGTTTCGAAGGCCGCC<br>ACGCACGAGTCATGGTAGCGCTGCCCGGCGACGGTGAGCCGGATGCGCTTGCCCTCTTTGAAGGTGAGCGAGAGGCCGAGAG<br>TGTCCTCAAGCAGCCGCATCTGTTGCGAGACCGCACCCGCGCTGATGTCGAGACGGCGGGCCGCCTCCACGATGCCACCGAA<br>ACGGCCCACGGCCTCGAAAACCTGGAGGGCGCGAAGCGACGGGAGGTTCATTATATATACCTCCTTTATTGTATCATGAGCG<br>GATACTTATGTCAAA
Promoter<br>*Pseudomonas* sp. 3A<br>P_{PS3A_06990}	GCGATGGCTATCGGGTCTATTCCTTGTCTTCGATGGCCATTCAGGCGGCGATTGCCGGGCAGGGCGTGGTCCTGGCGC<br>AATGCTCGATGATTACCGACGCCGTGGCAGACGGGCGTCTGGTCATTCCGTTCGCACAGGGGCTGAAGATGCCTTCAT<br>CGTACTTTCTGGTCGGGGCCAGGGATGCATTCGACAAGGCCCATTGCCGCGACTTCCATCGCTGGCTGGTGGCGCGCG<br>GGCGTGATCAGACAGTCATCAATGAGCAACTGTTGAGTCAGCCGTAAAACGTCACTCTTGTATGCGAAACACGCATA<br>GCAGCTAGAAACCGTGTCCGATTGTTGTGCGCAGGTGATGCAGCCACCATCCCACCATCGTTTGCGCCAACAACAAA<br>AAAGGATTCGAG-(ATG, PS3A_06990)
pNov	
Regulatory gene<br>*Novosphingobium* sp. 11B<br>NRB_04800	GACTCTAGAGGATCCCCGATTCACGCATAGCGAAACGGCTCCCGGCCGATGGCTTTTTCGGAGGTCCACGTATCGCAGACGGT<br>GCGGAAGGTGTCGACGAAGGCCGCCTGGAGGGCAGTCGGCCGCCAGTCTTCGCGGTACGTCAGCCCAATCTCGCGACGCAGG<br>CTGGAGCCTATATCACCGATTTCCGTGAGCAGCCCCGCGCGCTGGTCGAAGAGGAACTGGTCGCGCGACATCAGCGACAGCC<br>AATCTCCTTCGAGAAGGACGCCACGGACAACCAGGAGGCTGCCGCACTCGATGCGCAGGCGCGGGGGCTCCATACCGCGGTC<br>TATGAACATCTGCTCCCAACGCTGGCGGACCGGGGTCCCGCGGGCCGCAATAACCCAGGGGAAGTCCAGGAGCTTGTCGAAG<br>GACGCGTCGGTTCCCGACAGGGGGTGGCCGCTCCGCCCGACGATGACCGGGTCATCGGCGAAGAGGGGTTCCTGAACGACGT<br>CGGCGACGGGAAGCGGGTCGCGCATGGCCCCTATAAGGAGATCGCTCTCGCCCTGGCGGAGCATGCTGAGAAGCTCCAGATA<br>CGGGCCTTCGATCACGTTGACCGAGGCCGCCGGATAACGCTCGCTGAACAGGGCGAGGGCCTTGGGCAGGAGGATAGCCCGG<br>GAAAGCGGCATGGCGCAAACAATGAGGCGCCCGCCCTGCTTGGCGCCAATGCCCCGCAGTTCATCGAGCCCGGCGAGCAGCT<br>CGGACCGGGCAAGGCGGGCAAAGCGCAGGAAGCGGGCCGCAGCTGGCGTCGGCTGAACCGTCTTACCCTGACGCATCAGGA<br>GCGGGACCTCCATCTGTTGTTCGAGGTCCCGCGCCGACCGGTGCAGGGCCGGCTGGCTCAGACCAGTGTTCAGGCTCGCCAGC<br>GCGAAGCTCCCCGCATCGTCCACGGCGATAAGGGCGCGCAGCTGGCCCAGCGTAATACGGCGCTCGATATTCGGGAGGGGCG<br>ACAGGCGCGCGGCGCGCCGAACGGACCGACCGCCGCGCACTATATAGGCCAGGGCCCGTTCAATTCGAGTCGAGAGCAGTTC<br>GCCAGCTTCGGTCGCCACGGCGCCACCGGTTTGGCGAGCGAAGAGCGTGGTGCCAAGCTGGGCCTCGAGCTTGGACACGGCC<br>TGCGTGAGCGCCGGCTGCGACAGGTTCACAGCACCCGCGGCCGCGCTTATGGAACCCAGCCGGGTCGCGGCCGCGACCGCAT<br>TCAGGTGCCGCAGGTTGAGGTCCAGCGGGTCGATCATTATATATACCTCCTTTATTGTATCATGAGCGGATACTTATGTCAAA
Promoter<br>*Novosphingobium* sp. 11B<br>P_{NRB_04780}	GTCCGCCGAATGTCTGTTGTCGGCCTGCGCTGCAGGTGTCGAAGATGAGGTCATCGCTTCGCTCGACGCCAGCTTCCC<br>AGGCTGGGATTGGCGCGAGCGCGGCGATTACAATCTTGAGCGCATGCTCACGCATGGCGCCCGGCGTGCTGCCGAAA<br>TGCGCGACGAGTCCGCCATGACCGCACACGCGCTGGGGTGCGGCGGCGGCATGGCGACGGCGACTGCCGACTGGCA<br>GGATCGCCTGGGCGCACTTGCGATCACCCTGCTTCCAGGCGGCCTTGCCGGCAAGGCCGCCGCCATTCTTGCATCCGA<br>CGGAAAACACGCATC-(ATG, NRB_04780)

[a]The regulatory gene, *bla* promoter, Shine-Dalgarno sequence, and restriction enzyme site (XbaI) in the synthesized sequence are indicated in red, blue, green, and purple, respectively. The locus tag and start codon (ATG) of the downstream coding sequence are indicated at the 3' end of each promoter sequence.

Table S5. Monomer composition of softwood black liquor

Compounds[a]	Concentration
vanillin	20.4 mM
vanillic acid	5.73 mM
5-carboxyvanillin	2.44 mM
acetovanillone	2.00 mM
5-carboxyvanillic acid	0.85 mM
p-hydroxybenzaldehyde	0.29 mM
5-formylvanillin	0.16 mM
p-hydroxybenzoic acid	0.08 mM
5-formylvanillic acid	0.07 mM

[a]Data from Kamimura et al. [8].

Table S6. Taxonomic classification of the candidate strains

Strains	Class	Genus	Closest type strain	Identity[a]
8B	β-proteobacteria	*Cupriavidus*	*C. basilensis* DSM 11853	99.9%
3C	β-proteobacteria	*Burkholderia*	*B. gladioli* CFBP 2427	99.5%
2C	β-proteobacteria	*Paraburkholderia*	*P. diazotrophica* NKMU-JPY461	98.7%
3A	γ-proteobacteria	*Pseudomonas*	*P. bohemica* IA19	98.7%
11B	α-proteobacteria	*Novosphingobium*	*N. resinovorum* NCIMB 8767	99.8%
8C	Actinomycetia	*Pseudarthrobacter*	*P. enclensis* NIO-1008	98.4%

[a]Identity with the 16S rRNA gene sequences of the closest type strains.

Table S7. Summary of genome assembly statistics for the analyzed strains

Strains	Total length (bp)	No. of contigs	G+C content (%)	N50	No. of CDSs	No. of rRNA genes
Cupriavidus sp. 8B	11,311,448	679	64.3	81,482	10,166	1
Burkholderia sp. 3C	6,999,011	117	60.7	243,997	6,200	3
Paraburkholderia sp. 2C	7,457,045	89	63.3	391,422	6,578	2
Pseudomonas sp. 3A	7,747,894	163	66.8	210,954	6,544	2
Novosphingobium sp. 11B	6,092,655	350	65.3	45,575	5,299	2

Table S8. Genes identified in the vicinity of putative PDC hydrolase genes

Strains	Locus tag	Product	Most similar protein[a]	Identity[b]
Cupriavidus sp. 8B	CS8_000060	molybdate ABC transporter substrate-binding protein	PmdU (GAO68739.1)<br>4-oxalomesaconic acid tautomerase<br>*Comamonas* sp. E6	55.2%
	CS8_000070	hypothetical protein	PmdF (GAO68736.1)<br>4-carboxy-4-hydroxy-2-oxoadipic acid aldolase<br>*Comamonas* sp. E6	23.8%
	CS8_000080	dicarboxylate/amino acid:cation symporter	DctA (A0KAU6.1)<br>C4-dicarboxylate transport protein 1<br>*Burkholderia cenocepacia* HI2424	65.4%
	CS8_000090	PDC hydrolase	ProL (BAD04056.1)<br>PDC hydrolase<br>*Pseudomonas straminea*	45.2%
	CS8_000100	LysR family regulator	Nac (Q08597.1)<br>Nitrogen assimilation regulatory protein<br>*Klebsiella aerogenes*	29.2%
	CS8_000110	LysR family regulator	Nac (Q08597.1)<br>Nitrogen assimilation regulatory protein<br>*Klebsiella aerogenes*	31.0%
	CS8_000120	hypothetical protein	LigU (Q0KJL4.1)<br>(4E)-oxalomesaconate Delta-isomerase<br>*Novosphingobium* sp. KA1	9.2%
	CS8_000130	amidohydrolase family protein	PmdE (GAO68737.1)<br>4-oxalomesaconic acid hydratase<br>*Comamonas* sp. E6	77.8%
	CS8_000140	4-carboxy-4-hydroxy-2-oxoadipate aldolase/oxaloacetate decarboxylase	PmdF (GAO68736.1)<br>4-carboxy-4-hydroxy-2-oxoadipic acid aldolase<br>*Comamonas* sp. E6	62.4%
	CS8_000150	DUF1932 domain-containing protein	no hit	-
	CS8_000160	PDC hydrolase	ProL (BAD04056.1)<br>PDC hydrolase<br>*Pseudomonas straminea*	71.0%
Burkholderia sp. 3C	BGC_43250	LysR family regulator	Nac (Q08597.1)<br>Nitrogen assimilation regulatory protein<br>*Klebsiella aerogenes*	28.7%
	BGC_43260	PDC hydrolase	ProL (BAD04056.1)<br>PDC hydrolase<br>*Pseudomonas straminea*	46.1%
	BGC_43270	dicarboxylate/amino acid:cation symporter	DctA (B2AGL2.1)<br>C4-dicarboxylate transport protein<br>*Cupriavidus taiwanensis* LMG 19424	65.2%

Table S8. –*Continued*

Strains	Locus tag	Product	Most similar protein[a]	Identity[b]
Paraburkholderia sp. 2C	PBS_63060	LysR family regulator	Nac (Q08597.1)<br>Nitrogen assimilation regulatory protein<br>*Klebsiella aerogenes*	29.1%
	PBS_63070	PDC hydrolase	ProL (BAD04056.1)<br>PDC hydrolase<br>*Pseudomonas straminea*	44.6%
	PBS_63080	dicarboxylate/amino acid:cation symporter	DctA (B2AGL2.1)<br>C4-dicarboxylate transport protein<br>*Cupriavidus taiwanensis* LMG 19424	62.6%
	PBS_63090	porin	BP0840 (Q04064.1)<br>Outer membrane porin protein<br>*Bordetella pertussis* Tohama I	27.6%
	PBS_63100	molybdate ABC transporter substrate-binding protein	PmdU (GAO68739.1)<br>4-oxalomesaconic acid tautomerase<br>*Comamonas* sp. E6	55.8%
	PBS_63110	Rieske 2Fe-2S domain-containing protein	MdpJ (G8FRC5.1)<br>Rieske nonheme mononuclear iron oxygenase<br>*Aquincola tertiaricarbonis*	37.5%
	PBS_63120	PDR/VanB family oxidoreductase	VanB (AAN69333.1)<br>vanillic acid *O*-demethylase oxidoreductase<br>*Pseudomonas putida* KT2440	41.5%
Pseudomonas sp. 3A	PS3A_06940	aromatic ring-hydroxylating dioxygenase subunit alpha	VanA (AAN69332.1)<br>vanillic acid O-demethylase oxygenase subunit<br>*Pseudomonas putida* KT2440	33.5%
	PS3A_06950	hypothetical protein	no hit	-
	PS3A_06960	hypothetical protein	HisP (P07109.2)<br>Histidine/lysine/arginine/ornithine transport ATP-binding protein<br>*Escherichia coli* K-12	12.8%
	PS3A_06970	ABC transporter substrate-binding protein	ArgT (P09551.3)<br>Lysine/arginine/ornithine-binding periplasmic protein<br>*Escherichia coli* K-12	51.7%
	PS3A_06980	LysR family regulator	Gcv operon activator (P0A9F6.1)<br>Glycine cleavage system transcriptional activator<br>*Escherichia coli* K-12	30.3%
	PS3A_06990	PDC hydrolase	PcmC (AAK16526)<br>PDC hydrolase<br>*Arthrobacter keyseri*	45.5%
	PS3A_07000	dicarboxylate/amino acid:cation symporter	DctA1 (Q8Y2K5.1)<br>C4-dicarboxylate transport protein 1<br>*Ralstonia pseudosolanacearum* GMI1000	64.1%

Table S8. –*Continued*

Strains	Locus tag	Product	Most similar protein[a]	Identity[b]
Novosphingobium sp. 11B	NRB_04770	PDC hydrolase	PmdD (GAO68735.1)<br>PDC hydrolase<br>*Comamonas* sp. E6	67.2%
	NRB_04780	amidohydrolase family protein	LigJ (BAK65927.1)<br>4-oxalomesaconic acid hydratase<br>*Sphingobium* sp. SYK-6	74.3%
	NRB_04790	hypothetical protein	no hit	-
	NRB_04800	LysR family regulator	GalR (Q88JX7.1)<br>HTH-type transcriptional regulator<br>*Pseudomonas putida* KT2440	29.6%
	NRB_04810	4-carboxy-4-hydroxy-2-oxoadipate aldolase/oxaloacetate decarboxylase	LigK (BAK65930.1)<br>4-hydroxy-4-methyl-2-oxoadipic acid aldolase<br>*Sphingobium* sp. SYK-6	72.6%
	NRB_04820	4-oxalomesaconate tautomerase	LigU (BAK65931.1)<br>4-oxalomesaconic acid tautomerase<br>*Sphingobium* sp. SYK-6	70.9%
	NRB_04830	C4-dicarboxylate transporter DctA	DctA (C1D8S5.1)<br>C4-dicarboxylate transport protein<br>*Laribacter hongkongensis* HLHK9	51.8%

[a]Top-hit proteins identified by BLASTP searches against the UniProtKB/Swiss-Prot database. Accession numbers for the proteins are shown in parentheses.

[b]Amino acid sequence identity determined using the EMBOSS-Needle program (https://www.ebi.ac.uk/jdispatcher/psa/emboss_needle).

Table S9. Homology analysis of dicarboxylate symporters[a]

	BGC_43270	PBS_63080	PS3A_07000	NRB_04830	SLG_05590
CS8_000080	76.8%	76.0%	63.6%	50.0%	51.0%
BGC_43270		80.8%	64.5%	48.3%	51.0%
PBS_63080			64.8%	47.1%	48.6%
PS3A_07000				46.9%	47.8%
NRB_04830					45.0%

[a]Percent identity was calculated based on full-length amino acid sequence alignments using the Needle program. CS8_000080, *Cupriavidus* sp. 8B; BGC_43270, *Burkholderia* sp. 3C; PBS_63080, *Paraburkholderia* sp. 2C; PS3A_07000, *Pseudomonas* sp. 3A; NRB_04830, *Novosphingobium* sp. 11B; SLG_05590, *S. lignivorans* SYK-6.

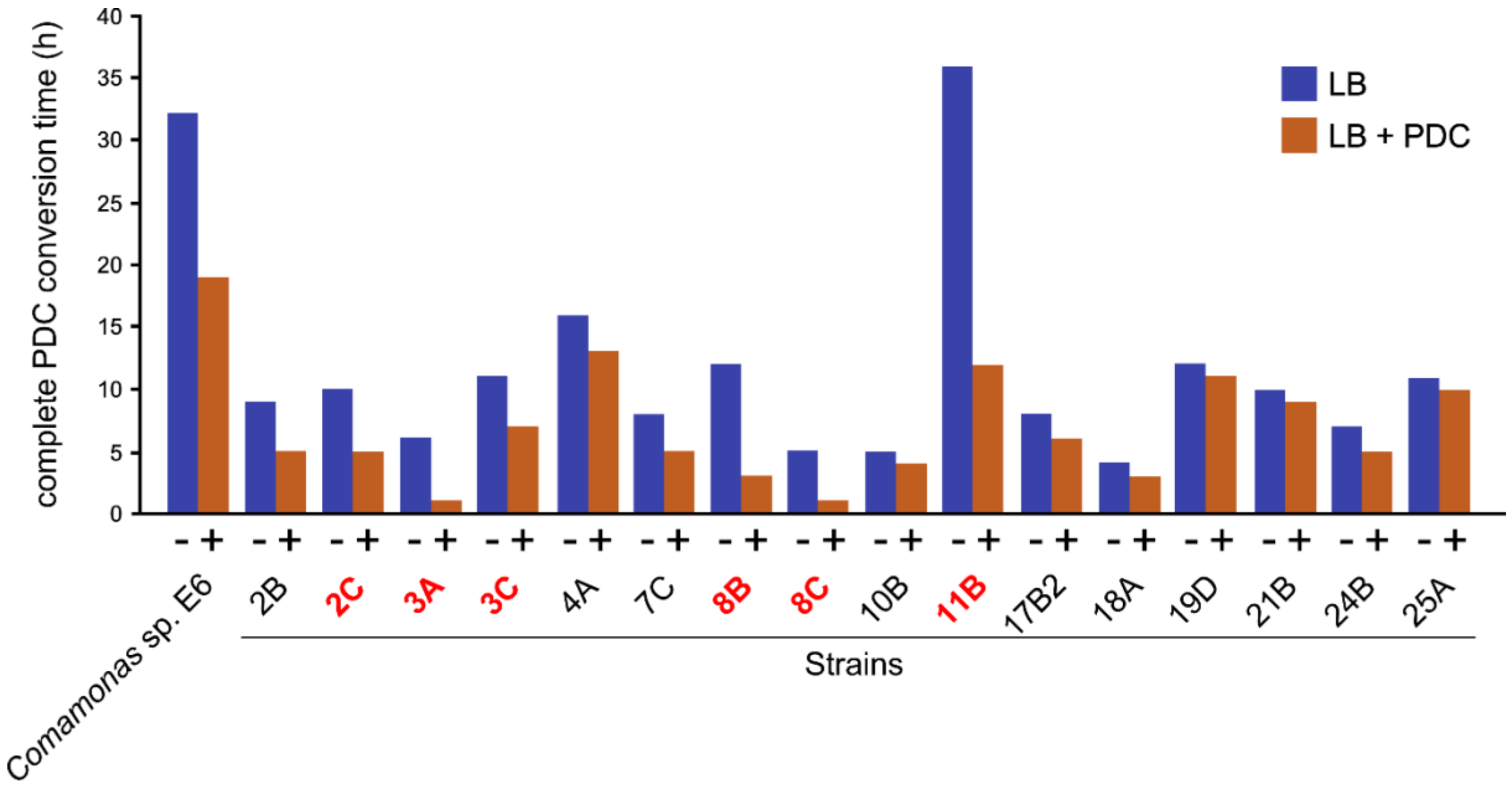

Fig. S1. Inducibility of PDC conversion activity in isolated bacteria.

Comamonas sp. E6, which exhibits known inducible PDC-converting activity, was included as a positive control. *Comamonas* sp. E6 and each isolate were cultured in LB (non-inducing conditions, −) or LB containing 10 mM PDC (inducing conditions, +), and resting cells were prepared and incubated with 500 µM PDC. The absorbance at 310 nm, the absorption maximum of PDC, was measured over time. The time required for the absorbance at 310 nm to reach a stable minimum was defined as the complete PDC conversion time. Results from a single experiment are shown for 16 strains in which the PDC conversion time was shortened under inducing conditions. The OD_{600} of resting cells was 0.1 for strains 2B, 2C, 4A, 7C, and 21B, and 0.2 for all other strains. Strains showing strong induction are highlighted in bold red.

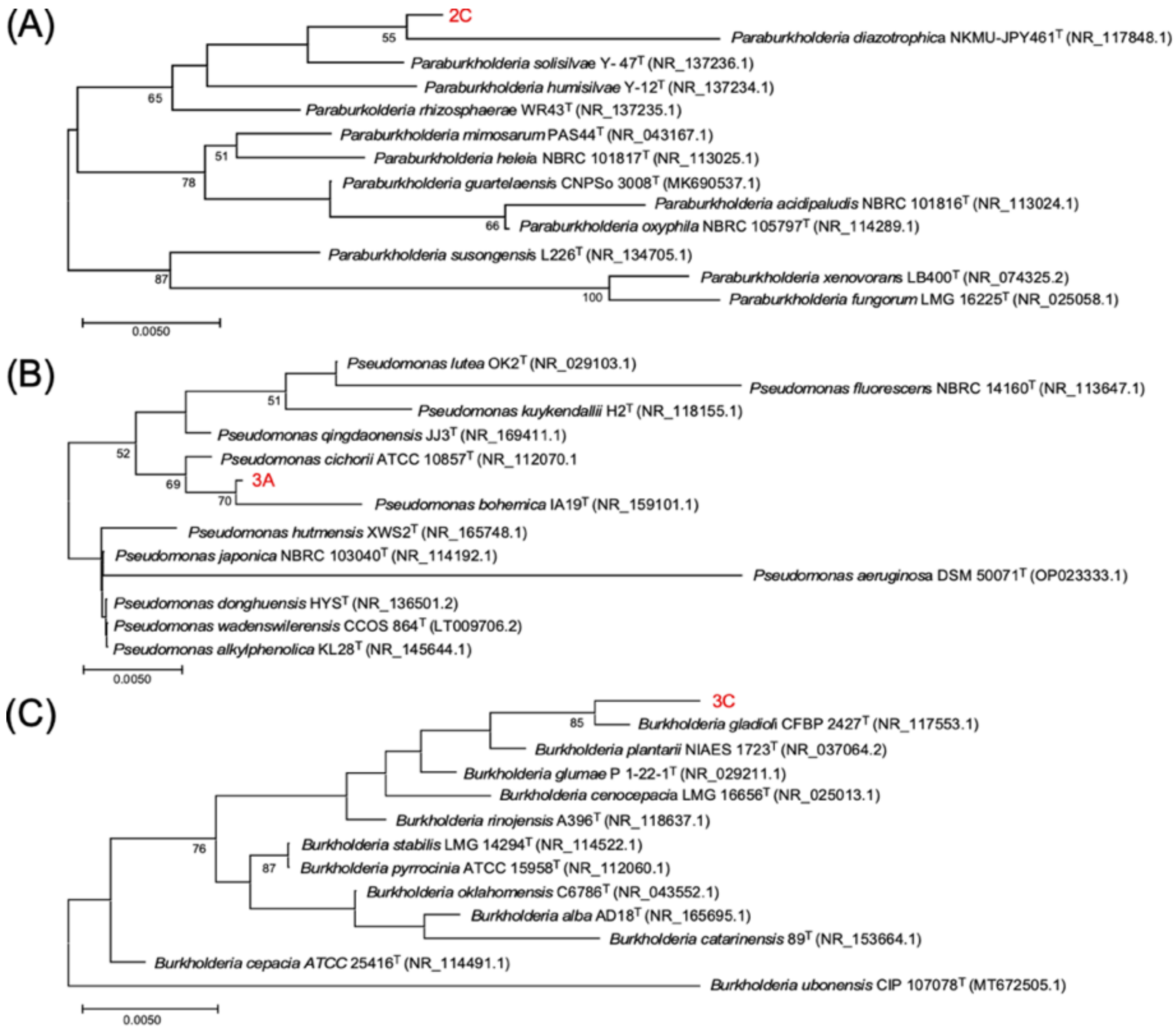

Fig. S2. Phylogenetic analysis of bacteria exhibiting inducible PDC conversion activity based on 16S rRNA gene sequences.

(A–F) Phylogenetic trees of strains 2C (A), 3A (B), 3C (C), 8B (D), 8C (E), and 11B (F). Phylogenetic trees were constructed by the maximum likelihood method based on the 16S rRNA gene sequences of each isolate together with those of the type strains of the top 10 most similar species identified by BLASTn searches against the NCBI 16S ribosomal RNA sequences (Bacteria and Archaea) database. Bootstrap values ≥50% are indicated at the branch points. Strains 2C, 3A, 3C, 8B, 8C, and 11B are shown in red. GenBank accession numbers are shown in parentheses, and type strains are indicated with a superscript "T".

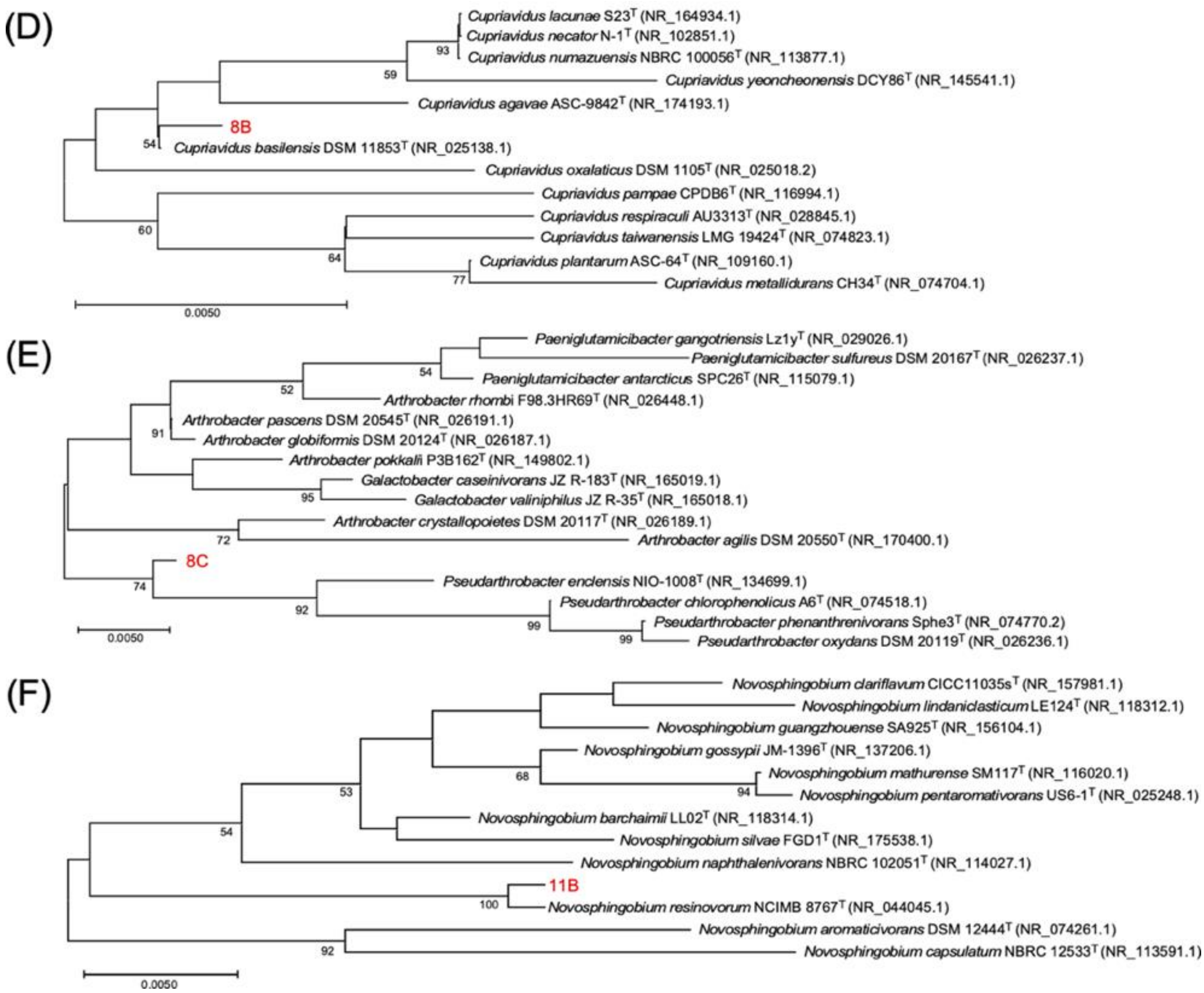

Fig. S2. -Continued

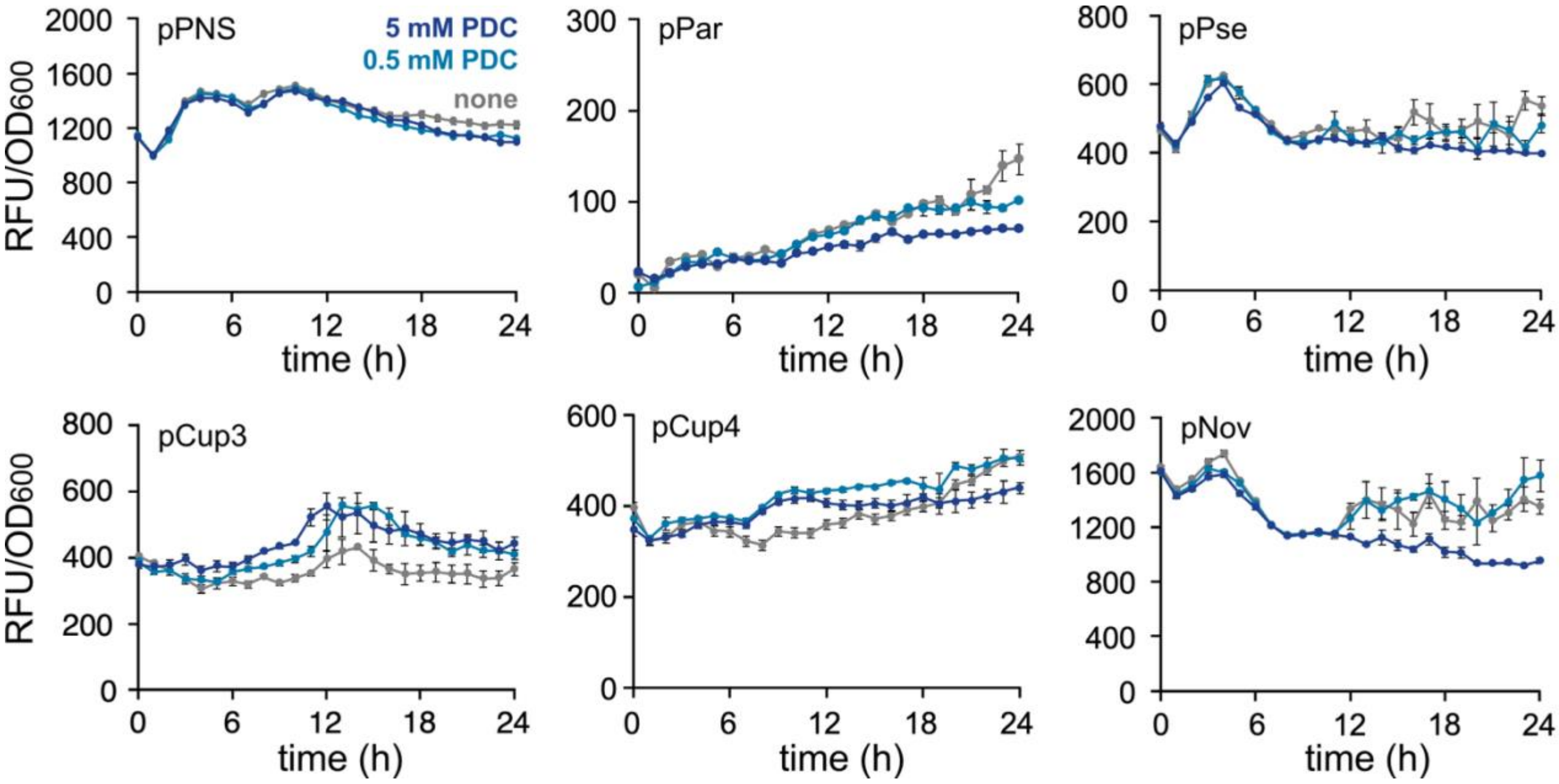

Fig. S3. Evaluation of PDC responsiveness.

Δ*ligI* harboring pPNS (vector control) or each candidate PDC biosensor plasmid (Table S4) was cultured in Wx-SEMP medium with or without 0.5 or 5 mM PDC. OD_{600} and mNeonGreen fluorescence (RFU) were measured over time, and RFU/OD_{600} was calculated at each time point. Each value represents the mean ± standard error (error bars) from three independent experiments.

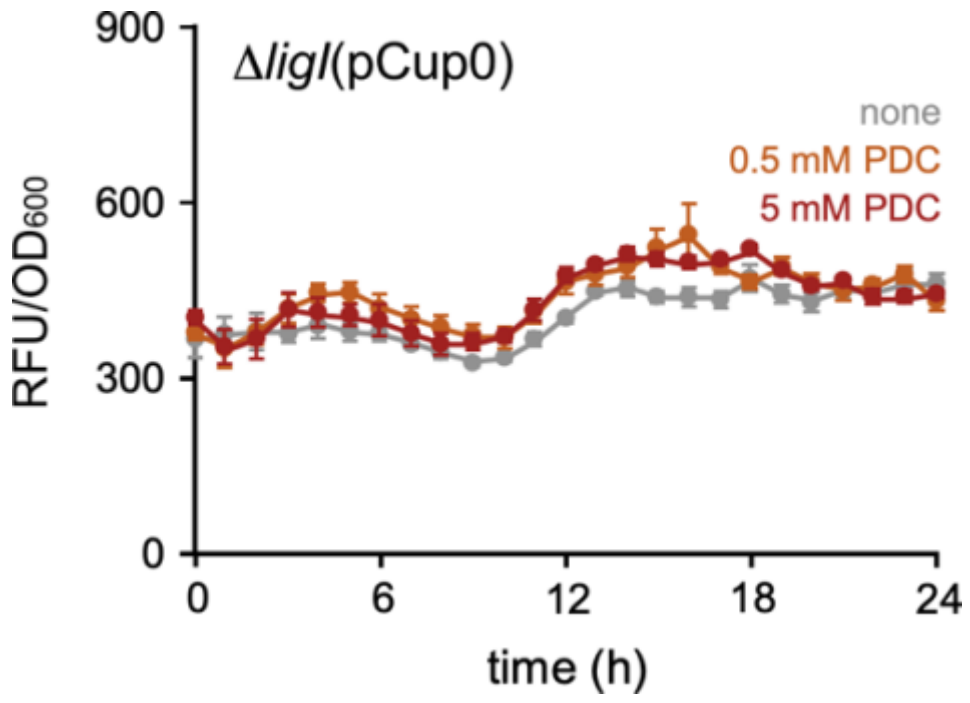

Fig. S4. Evaluation of PDC responsiveness in Δ*ligI*(pCup0).

Δ*ligI* harboring pCup0, a control plasmid containing only P_{PDC} without *pdcR*, was cultured in Wx-SEMP medium with or without 0.5 or 5 mM PDC. OD_{600} and mNeonGreen fluorescence (RFU) were measured over time, and RFU/OD_{600} was calculated at each time point. Each value represents the mean ± standard error (error bars) from three independent experiments.

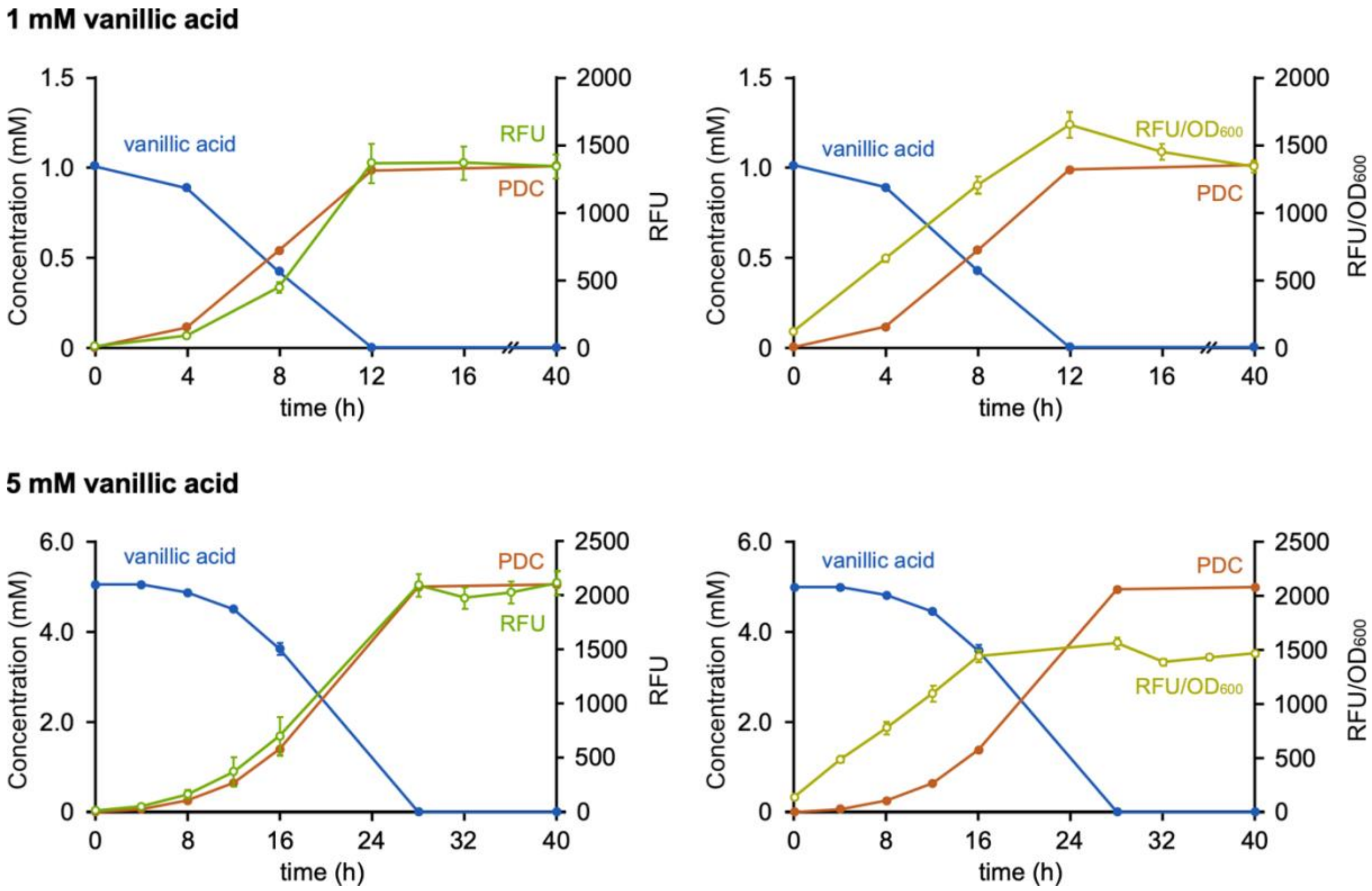

Fig. S5. Evaluation of the timing of PDC production and fluorescence response.

Δ*ligI*(pCup1) was cultured in Wx-SEMP medium containing 1 or 5 mM vanillic acid. The concentrations of vanillic acid and PDC were quantified by HPLC, while OD_{600} and mNeonGreen fluorescence (RFU) were measured using a microplate reader. RFU/OD_{600} was calculated at each time point. Each value represents the mean ± standard error (error bars) from three independent experiments.

References

1. Katayama Y, Nishikawa S, Nakamura M, Yano K, Yamasaki M, Morohoshi N, et al. Cloning and expression of *Pseudomonas paucimobilis* SYK-6 genes involved in the degradation of vanillate and protocatechuate in *P. putida*. Mokuzai Gakkaishi. 1987;33(1):77–79.
2. Mori K, Niinuma K, Fujita M, Kamimura N, Masai E. DdvK, a Novel Major Facilitator Superfamily Transporter Essential for 5,5′-Dehydrodivanillate Uptake by *Sphingobium* sp. Strain SYK-6. Appl Environ Microbiol. 2018;84(20):e01314–01318.
3. Sonoki T, Takahashi K, Sugita H, Hatamura M, Azuma Y, Sato T, et al. Glucose-Free *cis*,*cis*-Muconic Acid Production via New Metabolic Designs Corresponding to the Heterogeneity of Lignin. ACS Sustain Chem Eng. 2018;6(1):1256–1264.
4. Sasoh M, Masai E, Ishibashi S, Hara H, Kamimura N, Miyauchi K, et al. Characterization of the Terephthalate Degradation Genes of *Comamonas* sp. Strain E6. Appl Environ Microbiol. 2006;72(3):1825–1832.
5. Bolivar F, Backman K. Plasmids of *Escherichia coli* as cloning vectors. Methods Enzymol. 1979;68:245–267.
6. Figurski DH, Helinski DR. Replication of an origin-containing derivative of plasmid RK2 dependent on a plasmid function provided in trans. Proc Natl Acad Sci USA. 1979;76(4):1648–1652.
7. Martínez-García E, Fraile S, Algar E, Aparicio T, Velázquez E, Calles B, et al. SEVA 4.0: an update of the Standard European Vector Architecture database for advanced analysis and programming of bacterial phenotypes. Nucleic Acids Res. 2023;51(D1):D1558–D1567.
8. Kamimura N, Endo M, Nakamura B, Akiyama T, Kato R, Kawazoe M, et al. Platform Potential of *Sphingobium lignivorans* SYK-6 for Lignin Valorization via Biological Funneling. J Agric Food Chem. 2026;74(12):10406–10415.
